# Low wind, high impact: sustained low airflow alters water use efficiency and shapes plant acclimation

**DOI:** 10.1093/jxb/erag185

**Published:** 2026-04-17

**Authors:** Killian Dupont, Riccardo Frati, Silvère R M Vialet-Chabrand

**Affiliations:** Horticulture and Product Physiology (HPP), Wageningen University, PO Box 16, Wageningen 6700 AA, The Netherlands; Horticulture and Product Physiology (HPP), Wageningen University, PO Box 16, Wageningen 6700 AA, The Netherlands; University of Essex, UK

**Keywords:** Abaxial and adaxial side, boundary layer, microclimate, photosynthetic limitation, stomatal conductance, transpiration, water use efficiency

## Abstract

Minor differences in airflow are often assumed to have negligible physiological effects, yet they directly modify heat and gas exchange by altering the leaf boundary layer. We investigated how sustained low airflow impacts mechanisms regulating leaf–microclimate exchange, and how these responses shape acclimation in *Lactuca sativa* and *Solanum lycopersicum*. Lettuce was grown under two low-velocity airflow regimes (0.22 m s^−1^ and 0.65 m s^−1^), and changes in the leaf surface microclimate, plant transpiration, stomatal regulation, leaf photosynthesis, and growth were quantified. Increased airflow elevated transpirational demand, to which stomata responded dynamically by closing, increasing intrinsic water use efficiency. This closure did not fully offset enhanced water loss and was constrained by the need to maintain CO_2_ assimilation, which revealed that airflow alters a functional trade-off between carbon gain and water loss. This trade-off varied spatially, as photosynthetic limitation emerged specifically on the adaxial leaf side, indicating humidity-driven, side-specific diffusion limitations. At the whole-plant scale, increased airflow reduced fresh weight and leaf length by ∼13%, associated with hydraulic constraints and thermal regulation. We propose a unifying theory in which physiological regulation to airflow constrains structural and leaf-level acclimation, setting limits for plant growth. This framework provides a mechanistic understanding of how airflow affects short-term feedback and acclimation, with important consequences for experimental interpretation and reproducibility.

## Teaser

Sustained low airflow shifted the balance between CO_2_ uptake and H_2_O loss: stomatal closure increased iWUE but could not prevent transpiration-driven constraints on growth, revealing how airflow affects the microclimate, short-term feedback regulation, and acclimation.

## Introduction

Airflow plays a dual role in plant physiology, affecting both plant structure and the within-canopy microclimate. Mechanically, airflow-induced movement can generate dynamic light environments in field-grown crops and can trigger thigmomorphogenesis, a developmental acclimation that often results in more compact, sturdier plants (Braam, 2005; Burgess *et al*., 2019; Bauer and Poppinga, 2022; Brenya *et al*., 2022; Durand *et al*., 2026). Simultaneously, airflow stimulates exchange processes in the short term across leaf and canopy scales, modifying the microclimate of the plant (Jarvis and McNaughton, 1986; Schuepp, 1993). At low airflow velocities (0–1 m s^−1^), plant movement is minimal, so responses are driven more by microclimatic changes than structural acclimation due to physical movement (Smith and Ennos, 2003; Anten *et al*., 2010). However, most literature about airflow has focused on mechanical effects due to its predominance in the field, while diffusive or convective effects that dominate in low airflow environments were less studied (Ono *et al*., 2024, Preprint; Dupont *et al*., 2025).

In controlled environment agriculture (CEA), airflow velocities can range from 0.05 m s^−1^ to 1 m s^−1^, with recommendations typically ∼0.3 m s^−1^ to 0.5 m s^−1^, although it is unclear where to measure it (Frantz *et al*., 2004; Poorter *et al*., 2016; Peiro *et al*., 2020; Carpineti *et al*., 2024; Tarr and Lopez, 2025; Vincent *et al*., 2025). Because exchange from the leaf increases non-linearly in response to air velocity, small changes at low airflow can have disproportionately large effects: for example, raising airflow from 0.2 m s^−1^ to 0.5 m s^−1^ increases diffusive exchange due to reducing the leaf boundary layer by ∼58%, whereas a further increase from 0.5 m s^−1^ to 0.8 m s^−1^ enhances it by only ∼26% (Jones, 2013). However, airflow is rarely reported despite ample variation between facilities and differences in control strategies. A tacit assumption is that airflow variations within this range have minimal impact on plant growth and development, which may not hold. One piece of evidence is that small airflow variations could alleviate transpiration-related disorders such as tip burn in lettuce (*Lactuca sativa*) and blossom-end rot in tomato (*Solanum lycopersicum*) (Frantz *et al*., 2004; Heuvelink, 2018). Previous literature also suggested that even small increases in the low airflow velocity range (e.g. from 0.1 m s^−1^ to 0.5 m s^−1^) had phenotypic impacts (Wadsworth, 1959; Smith and Ennos, 2003; Frantz *et al*., 2004; Anten *et al*., 2010; Carvalho *et al*., 2015; Zhang *et al*., 2025). However, inconsistent experimental conditions (e.g. timing, intensity, and duration of treatments), in some cases combined with insufficient replication, prevent comparison between studies. Additionally, generalizations remain difficult because many studies do not provide a comprehensive understanding of the underlying mechanisms determining plant functioning in response to low airflow.

Airflow impacts plant–environment interactions jointly via the physical mechanisms of diffusion and convection, and their combined effects are typically lumped into apparent conductances. Following this convention, exchange is governed by the aerodynamic conductance (*g*_a_), boundary-layer conductance (*g*_b_), and stomatal conductance (*g*_s_) which operate in series at the canopy, leaf surface, and stomatal pore, respectively. Increases in gas and heat exchange induce microclimatic variations that alter photosynthesis, transpiration, and leaf energy balance, to which the plant may respond (Collatz *et al*., 1991; Schymanski and Or, 2016; Kimura *et al*., 2024; Dupont *et al*., 2025). Hence, the simple physical expectation that airflow increases exchange is not sufficient to predict plant growth and development because the interaction between responses, feedback regulation (e.g. by the stomata), and acclimation to airflow could determine the plant phenotype under low airflow.

The leaf-to-air coupling framework provides a potential conceptual foundation for interpreting plant–air interactions (Jarvis and McNaughton, 1986; Tardieu and Simonneau, 1998; Chelle, 2005; Condon, 2020). The leaf does not experience the surrounding air directly. Instead, a diffusive boundary layer surrounds the leaf, creating a microclimate that can differ substantially from ambient conditions (Jarvis and McNaughton, 1986; Schuepp, 1993). Increasing airflow strengthens the leaf-to-air coupling by thinning the boundary layer and enhancing exchange processes. Under low airflow, however, small differences in airflow can generate heterogeneous microclimates around leaves and within canopies, despite identical above-canopy air, which in itself displays large variability (Savvides *et al*., 2013, 2017; Kimura *et al*., 2020a, b; Plas *et al*., 2025). The leaf-to-air coupling may even differ between the adaxial and abaxial leaf sides due to variations in stomatal distribution and boundary layer properties (Foster and Smith, 1986; Fanourakis *et al*., 2015; Schymanski and Or, 2017; Wall *et al*., 2022). For example, the abaxial side may be more sheltered from airflow and experience less air exchange than the adaxial side, potentially leading to less responsive stomata (Carvalho *et al*., 2015). This spatial heterogeneity is important because it determines local plant responses and is thereby necessary for prediction of the whole-plant response (Chelle, 2005; De Kroon *et al*., 2005; Savvides *et al*., 2013). However, the degree to which low airflow alters microclimatic gradients remains poorly quantified at different scales, such as close to the leaf surface (Kimura *et al*., 2020b).

Spatial heterogeneity in the leaf microclimate, such as vapour pressure, temperature, and CO_2_ gradients, governs transpiration and photosynthesis, and elicits stomatal feedback regulation. A feedback response of the stomata under an increase in airflow can occur in the short term (minutes to hours) due to the increase in leaf-to-air coupling, and leads to stronger stomatal control of transpiration, since stomata are better connected to the ambient microclimate. In contrast, under weak coupling (low airflow), transpiration is dominated by external conditions (Jarvis and McNaughton, 1986). Modelling studies demonstrated that increasing air velocity <1 m s^−1^ could increase the vapour pressure deficit at the stomatal pore (VPD_s_) by up to 1 kPa, and could reduce leaf temperature by as much as 6 °C, depending on species and environmental context (Collatz *et al*., 1991; Dupont *et al*., 2025). These changes trigger rapid stomatal adjustments within minutes, including feedback to altered transpiration, which result from enhanced exchange processes and altered leaf-to-air vapour gradients (Kuiper, 1961; Renard and Demessemacker, 1983; Dixon and Grace, 1984; Bunce, 1985; Collatz *et al*., 1991; Aphalo and Jarvis, 1993; Tardieu and Simonneau, 1998; Schymanski and Or, 2016; Grossiord *et al*., 2020; Shapira *et al*., 2024). However, these studies focused on short-term responses and regulation, and did not capture exposure to sustained wind under realistic growth conditions, the subsequent impact on plant water balance, and acclimation to low airflow over days to weeks (Buckley *et al*., 2011; Schymanski and Or, 2016; Still *et al*., 2019).

Expansive growth relies on water movement driven by water potential differences, and may decline when transpiration reduces water availability in growing tissues (Barillot *et al*., 2021; De Swaef *et al*., 2022). Stomatal feedback regulation can stabilize the water potential gradient by reducing water loss, but such regulation alone does not always compensate for sustained increases in evaporative demand, since stomata also respond to other environmental stimuli (Buckley, 2017, 2019; Lawson and Vialet-Chabrand, 2019). Plants have to balance water loss and carbon gain, and may progressively shift towards anatomical adjustments that lower transpiration when evaporative demand increases, such as under increased VPD_leaf_ (Carins Murphy *et al*., 2014; Tardieu *et al*., 2015; Amitrano *et al*., 2019; Du *et al*., 2019; Grossiord *et al*., 2020; Koehler *et al*., 2023). An example is differences in leaf elongation due to altered transpiration under a decrease in humidity, such as the ∼20% decrease in elongation observed under a 0.80 kPa decrease in Barillot *et al*. (2021). However, it remains unclear whether subtle differences in low airflows cause similar acclimation. Additionally, any change in transpiration impacts the leaf temperature, in addition to sensible heat exchange, which can in turn affect temperature-dependent developmental processes such as leaf initiation (Savvides *et al*., 2016; Heuvelink, 2018). This may lead to a complex interplay between stomatal regulation, water status, leaf temperature, transpiration, and growth.

We therefore aimed to answer how minor differences in the low airflow velocity range (0–1 m s^−1^) impact the water–carbon trade-off at the leaf and plant scale and what the consequences are on plant growth. We hypothesized that sustained low airflow shapes the relative contribution of stomatal feedback regulation and longer-term leaf- and plant-level acclimation. The leaf–air coupling theory provides a mechanistic basis for understanding how airflow regulates transpiration, temperature, and stomatal behaviour on short time scales and its impact on the leaf surface microclimate. However, how different couplings impact acclimation mechanisms to shape whole-plant growth remains unresolved. To minimize mechanical effects, we imposed two low airflow velocities (0.22 m s^–1^ and 0.65 m s^−1^) during growth, to quantify their impact on the plant phenotype, through their influence on exchange processes.

## Materials and methods

### Plant material and germination

Lettuce (*Lactuca sativa*) serves as a model plant for this study due to its importance in CEA research (van Delden *et al*., 2021; Kaiser *et al*., 2024) and its compact growth form, consisting of transpiring leaves, suggesting that heat and gas exchange responses to the environment can be observed with minimal mechanical effects (Anten *et al*., 2010). An additional experiment with a subset of measurements (leaf temperature, leaf gas exchange, early leaf expansion, and fruit harvest) was performed with dwarf tomato plants (*Solanum lycopersicum*) under similar growth conditions to assess the generality of some of the lettuce responses observed in another crop species.

Lettuce seeds (*L. sativa* ‘Cecilia’, Rijk Zwaan, De Lier, The Netherlands) were sown on rockwool plugs (2.2×2.7 cm, Grodan, Roermond, The Netherlands) and covered with vermiculite (Pull BV, Rhenen, The Netherlands). The plugs were fully saturated with distilled water before being placed in the dark at 4 °C for 2 d. Next, seedlings were exposed to light for 5 d before transplantation. Uniform seedlings with dicots were selected and transplanted in rockwool cubes of 7.5 cm width and 6.5 cm height (Plantop, Grodan, Roermond, The Netherlands), covered with white plastic to prevent algal growth and excessive evaporation from the cube. These were then placed in four rows with 7.5 cm spacing between the rows, for a total of 28 plants per compartment, resulting in 85 plants m^–2^. Tomato seeds (*S. lycopersicum* ‘Plum Tomato Red’, Vreugdenhil, De Lier, The Netherlands) were germinated using the same germination protocol, and seedlings were transplanted after 22 d into rockwool cubes of 10 cm width and 6.5 cm height, covered with white plastic. A total of 12 plants per compartment were used, placed at a density of 30 plants m^−2^.

### Base and enhanced airflow treatment

Throughout this manuscript, we use the term ‘airflow’ to describe forced air movement within the growth compartments. Although ‘wind’ comprises both horizontal and vertical components in meteorological terms, it is most commonly associated with horizontal atmospheric air movement. In lettuce, the imposed airflow was primarily horizontal and could resemble wind, whereas in tomato airflow was applied both horizontally and vertically. We therefore use airflow as a broader term encompassing both horizontal and vertical air velocities within controlled environments. This terminology also distinguishes chamber airflow from the imposed flow rate within a gas exchange cuvette.

#### Butterhead lettuce

After transplanting, lettuce seedlings were randomly assigned to one of the two low airflow treatments within the same climate chamber: the base horizontal air velocity of 0.22 m s^−1^ (airflow–) and an enhanced horizontal velocity of 0.65 m s^−1^ (airflow+). The chamber was divided into two compartments, with treatments alternated between compartments across repetitions to account for spatial variability. The airflow– treatment was present due to the inflow rate of the horizontal recirculating airflow system, as part of the climate control of the growth chamber. To create the airflow+ treatment, a 4×3 grid of 12 computer fans (Noctua NF12 PWM chromax.black.swap 120 mm fan, Rascom Computer distribution, Vienna, Austria) were installed to draw air horizontally across the canopy within the compartment (Supplementary Fig. S1). Manufacturer specifications indicated a maximum airflow of 93.4 m^3^ h^−1^ and static pressure of 2.61 mm H_2_O. Fans were secured without spacing using zip ties to ensure a uniform flow field and operated continuously at 1500 rpm via a PWM controller (NA-FC1, Noctua). This ensured that, despite minor fluctuations in the chamber’s baseline airflow between repetitions, airflow+ always provided a consistent increase relative to airflow–. This resulted in an upper velocity range that partially removed the boundary layer, without inducing adverse mechanical effects. The airflow velocities chosen were typical for growth chambers and vertical farms (Frantz *et al*., 2004; Peiro *et al*., 2020; Carpineti *et al*., 2024).

Horizontal air velocity was measured at 10 cm height using a NIST-calibrated hot-wire anemometer (8475, TSI Incorporated, Shoreview, MN, USA) in an empty chamber. Measured velocities averaged 0.22 m s^−1^ (range 0.13–0.30 m s^−1^) for airflow– and 0.65 m s^−1^ for airflow+ (range 0.535–0.77 m s^–1^) between repetitions. The spatial uniformity range (maximum–minimum value) was 0.10 for airflow– and 0.21 for airflow+ (Supplementary Fig. S2). The stability of the base airflow was measured with a 2D sonic anemometer (Atmos 22, Meter Group, München, Germany), logging every 5 min, and placed near the air outlet. While this flow was relatively stable, the minor fluctuations may explain some of the differences between repetitions in time (Supplementary Fig. S3).

#### Dwarf tomato

After transplanting, seedlings were randomly assigned to a total of 12 compartments (0.80×0.50 m), each with 12 plants. Compartments were equally divided between the left and right sides of the room, to account for potential spatial effects. In the base airflow (airflow–), plants experienced the horizontal background airflow of the growth room. In contrast to lettuce, the enhanced airflow treatment (airflow+) in tomato was created by exposing only the two centre plants to an additional vertical airflow supplied by a inline duct fan positioned between the lights above the canopy, pointing towards the plant (Model HI-100SD, Hon&Guan, Guangdong Province, China). This choice was deliberately made because dense border plants in a tomato canopy would have caused substantial obstruction of a horizontal flow. As the plants grew in height towards the fan, the speed of the fans was adjusted to maintain a similar air velocity around the meristem. This resulted in airflow– of 0.21 m s^−1^ and airflow+ of 1.03 m s^−1^.

### Growth conditions and microclimate measurement

#### Butterhead lettuce

Lettuce plants were watered twice daily with a 2.3 dS m^−1^ nutrient solution containing (in mmol l^−1^): $0.38\;{\text{NH}}_{4}^{+}$, 8.5 K^+^, 4.1 Ca^2+^, 1.15 Mg^2+^, $12.8\;{\text{NO}}_{3}^{-}$, 1.6 Cl^−^, $1.65\;{\mathrm{SO}}_{4}^{2-}$, $1.65\;H_{2}{\mathrm{PO}}_{4}^{-}$; and (in μmol l^−1^): 0.38 Si, 3.8 Mn, 3.8 Zn, 38.3 B, 0.77 Cu, 0.38 Mo, 31 Fe through ebb and flow irrigation. Plants were grown under controlled conditions with a light intensity of 180 µmol m^−2^ s^−1^ photosynthetic photon flux density (PPFD) (Supplementary Fig. S4), measured with a LI-250A light meter (LI-COR Biosciences, Lincoln, NE, USA). The spectral distribution was 10.3% blue; 17.3% green; 72.3% red, measured with an LI-180 spectrometer (Supplementary Fig. S5). Together with a 18/6 h light/dark photoperiod, this resulted in a daily light integral (DLI) of 11.66 mol, and temperatures of 22/20 °C during the day and night. The night temperature setpoint started 1 h before the end of the photoperiod, which led to a gradual decrease to 20 °C by the end of the photoperiod. [CO_2_] was set to a minimum of 450 μmol mol^−1^ and relative humidity to 75%, and resulted in a VPD_air_ of 0.66 kPa during the photoperiod and 0.59 kPa during the night.

The resulting above-canopy air temperature and humidity under both treatments were measured with a temperature and humidity sensor covered in aluminium to shield from radiation (Hobo MX2301A, HOBO Data loggers, MI, USA). Accumulated thermal time over the experimental period was calculated using a base temperature of 4 °C, based on replicate-weighted mean air temperatures, and accumulated over the 26.5 days after transplant (DAT), to evaluate potential differences in temperature-driven development between treatments (Stanghellini *et al*., 2024; Paredes *et al*., 2025). A miniature humidity and temperature sensor (SEK-SHT40, Sensirion AG, Stäfa, Switzerland) was additionally used to measure the microclimate (humidity and temperature) near the plant at 24–25 DAT. Two sensors were taped together, with one oriented adaxially and the other abaxially, leaving an ∼5–6 mm gap to position the leaf between them. Sensors were placed 3.5 cm from the leaf tip, and six plants per treatment were measured on the last fully expanded leaf. Leaf temperature was determined on a light-exposed leaf using an infrared thermometer (Raynger ST, Raytek Corporation, CA, USA) with emissivity set to 0.95. Meristem temperature was approximated by measuring the temperature of the centre of the plant.

#### Dwarf tomato

Plants were watered every other day using ebb and flow with nutrient solution at 2.1 dS m^−1^, consisting of macronutrients (in mmol l^−1^) 1.2 NH_4_^+^, 7.2 K^+^, 4.0 Ca^2+^, 1.82 Mg^2+^, $12.4\;{\text{NO}}_{3}^{-}$, $3.32\;{\mathrm{SO}}_{4}^{2-}$, 1.0 P; and micronutrients (in μmol l^−1^) 35 Fe, 8 Mn, 5 Zn, 20 B, 0.5 Cu, and 1.5 Mo. Side shoots were pruned weekly, plants were supported by a vertical stake starting at 28 DAT, and flowers were pollinated 2–3 times per week. Fruits were harvested at 70 DAT. Light was provided by Philips GreenPower LED DR/W 150 modules (Signify, Eindhoven, The Netherlands), delivering 165 μmol m^−2^ s^−1^ at 7 cm height (DLI 9.5 mol m^−2^ d^−1^) and 230 μmol m^−2^ s^−1^ at 25 cm height (DLI 16 mol m^−2^ d^−1^). The spectral distribution consisted of 3.5% between 400 nm and 500 nm, 11.1% between 500 nm and 600 nm, 83.97% between 600 nm and 700 nm, and 1.41% between 700 nm and 800 nm. Air temperature of the room was set to 23 °C during the photoperiod and 20 °C during the night, humidity was constant at 70%, and CO_2_ was maintained at a minimum of 400 μmol mol^−1^. Above-canopy air temperatures and humidity were measured 5 cm above the meristem with a shielded miniature sensor (SEK-SHT40, Sensirion AG) and leaf temperature using the infrared thermometer.

### Morphological measurements

#### Ground coverage and final harvest

Top-view pictures of lettuce were taken every 30 min throughout the photoperiod by two cameras per treatment (Raspberry PI camera module V3, Raspberry Pi Foundation, Caldecote, UK) to obtain canopy ground coverage, by using HSV segmentation and a Richards growth curve fitted to time-series data (Supplementary Protocol S1). At 26.5 DAT, plants were harvested, and leaf number was determined by counting all leaves >1 cm in length. Tip burn incidence was scored as present or absent, defined by any visible discoloration within the crown. Fresh weight was determined, and total leaf area was measured using a leaf area meter (LI-3100C, LI-COR Biosciences). Samples were subsequently dried in a forced-air oven at 105 °C for 2 d to obtain dry weight. Specific leaf area (SLA) was calculated as the ratio of leaf area to dry weight.

#### Stomatal imprints

Stomatal imprints of lettuce leaves were obtained using silicone dental impression material (Elite HD+, Zhermack, Badia Polesine, Italy) applied to the leaf surface with a silicone gun. From four plants per treatment for each replication, three imprints from the adaxial and abaxial sides were taken. Once hardened, clear acrylic nail polish was applied to each imprint, allowed to dry, and then peeled off and placed on a microscope slide, magnified to ×25 using an optical microscope (Leitz Aristoplan, Ernst Leitz, Rijswijk, The Netherlands), and imaged with a digital camera (Axiocamera 305 Color, Carl Zeiss, München, Germany). Screenshots were taken and then analysed with a Python script that sequentially displayed images, which allowed the user to mark the stomata, and recorded the counts to an Excel file. Stomatal density was calculated as the number of stomata per image area, with image size converted from pixels to mm^2^ using a calibration factor of 0.18 µm px^−1^.

#### Leaf cross-sections and airspace

Cross-sections of three lettuce plants per treatment were taken for each replication, taking two samples per plant from the last fully expanded leaf, and fixed in resin using the protocol described in Shrestha *et al*. (2025). Samples were then cut using a microtome (Reichert-Jung 2050; R. Jung, Vienna, Austria) into 5, 10, and 20 μm thick slices, placed on slides, stained using 1% filtered toluidine blue, and rinsed with water to remove the excess, after which they were imaged at ×25, ×50, and ×80 magnification. The stained regions were defined as non-airspace and the unstained regions as airspace, though we acknowledge that cutting cells in half could have led to some misclassification, where a partially cut cell appeared as airspace. Leaf cross-sections were segmented in CIELAB space, with adaxial/abaxial sides assigned based on airspace proportion and verified visually (Supplementary Protocol S2). Because thresholding can influence absolute magnitudes, we focused solely on relative comparisons between treatments. A thickness of 10 μm was selected as the optimal slice thickness for airspace estimation because the reduction in airspace between the 10 μm and 5 μm slices plateaued, which indicated that further sectioning (to 5 μm) revealed no additional information relevant to airspace quantification. Thus, the 10 μm slices were deemed to best capture the internal tissue structure while minimizing the inclusion of multiple cell layers overlapping.

### Plant transpiration measurement

Whole-plant lettuce transpiration was measured gravimetrically under the respective airflow treatments for three plants per treatment for each replication 21–25 DAT. Plants were taken from the canopy, and the rockwool cubes were wrapped with plastic wrap to prevent water loss from the substrate. The plant was then placed on a precision balance (2200 g capacity; FPRS2202, Fisher Scientific, Landsmeer, The Netherlands) positioned within the growth environment, ensuring similar PPFD and airflow velocity to that at the original canopy positions. Weight loss was logged every 5 s over the photoperiod and the subsequent night. After transpiration measurements, total leaf area of each plant was determined, and whole-plant transpiration rates were expressed both per plant and per unit leaf area (mmol H_2_O m^−2^ s^−1^).

Raw data were processed to remove transient artefacts by identifying short-term weight spikes caused by external disturbances (e.g. vibration) as outliers based on deviation from a locally smoothed trend (LOESS, span=0.3). The 95% of points closest to the fitted trend were retained, removing only the most extreme outliers. Start weight was defined as the mean of the first five readings, and transpiration rates (*E*) were obtained as the slope of the cumulative weight loss versus time, fitted separately for the day and night period. Preliminary tests with a constant reference weight over 36 h confirmed that there was no balance drift.

### Leaf gas exchange measurements

Net photosynthetic rate (*A*) and *E* were measured on the last fully expanded leaf with a portable gas exchange system (LI-6800, LI-COR Biosciences) equipped with the 6800-01A fluorometer light source and 2 cm^2^ chamber aperture. Intrinsic water use efficiency (iWUE) was calculated as the ratio of *A* to *g*_sw_. Cuvette setpoints were selected to match the growth environment of the plants as closely as possible, except for the airflow rate, which nearly eliminates the boundary layer within the leaf cuvette (fan speed 10 000 rpm, flow rate 500 µmol s^−1^). Plants from different treatments were measured in alternating order to minimize potential time-of-day effects. The infrared gas analysers were matched before the first measurement and subsequently re-matched every 30 min, or whenever a substantial humidity change occurred.

#### Snapshot measurement in the growth environment

To quantify the operational *g*_sw_ to sustained low-airflow conditions, we conducted snapshot measurements of leaf gas exchange (26 DAT for lettuce, 35 DAT for tomato) on six plants of each treatment per replication for lettuce, and two plants of each treatment per replication for tomato. During the snapshots, cuvette conditions were maintained at 450 µmol mol^−1^ CO_2_, 75% relative humidity, 22 °C air temperature, and 185 µmol m^−2^ s^−1^ incident PPFD. Daytime and nocturnal gas exchange was recorded at four time points: 1 h before the onset of the photoperiod, and 1, 4, and 7 h after its start.

Data were logged once *A* had stabilized (∼1.5–2 min); however, because *g*_sw_ responds slowly, rather than waiting for *g*_sw_ stabilization, measuring quickly ensured that *g*_sw_ remained representative of the growth environment. Consequently, *A*, which responds rapidly, represented steady-state assimilation under cuvette conditions (thin boundary layer, mean *g*_bw_=3.34 mol m^−2^ s^−1^, SE=0.31) as influenced by *g*_sw_. In contrast, *g*_sw_ captured the operational stomatal behaviour in the low-airflow growth environment, as influenced by gradients that may develop, and avoided substantial short-term feedback responses to airflow as observed previously by Schymanski and Or (2016) and Shapira *et al*. (2024). These measurements therefore quantified *g*_sw_ as a trait determined by prior airflow conditions and allowed for separation of the relative contribution of *g*_sw_ and *g*_bw_ on the total conductance to water vapour. This additionally allowed evaluation of how biological control on transpiration by the stomata changes under sustained airflow.

To assess the influence of *g*_b_ representative of the growth environment, theoretical assimilation (*A*_theory_) and transpiration (*E*_theory_leaf_) rates were recalculated using Fick’s law. The LI-6800 cuvette *g*_b_ was replaced by the environmental *g*_b_ measured in the growth environment. For CO_2_, total conductance was calculated as the series combination of *g*_bc_ and *g*_sc_, and *A*_theory_ was estimated assuming a constant *C*_i_/*C*_a_ ratio. Similarly, for water vapour, transpiration was reconstructed using measured VPD_leaf_ and total conductance to H_2_O. Theoretical instantaneous water use efficiency (instWUE_theory_) was then calculated as the ratio of *A*_theory_ to *E*_theory_leaf_.

#### Abaxial and adaxial vapour pressure deficit response

To characterize the impact of increased diffusive gradients on the lettuce leaf gas exchange and its interaction with acclimation and the leaf side (abaxial or adaxial), *A* and *E* responses to a VPD_air_ step change were measured using the LI-6800 split-chamber system (part number 9968-313; LI-COR Biosciences). This approach increased the total diffusive gradient and ease of diffusion experienced by the leaf, determined by *g*_sw_, *g*_bw_, and VPD_leaf_, beyond that present in the growth environment. The change in VPD_air_ was achieved by altering humidity while air temperature was kept constant. Because the air surrounding the leaf was controlled in the portable gas exchange system, changes in *E* were driven primarily by changes in *g*_sw_ and not much by changes in leaf temperature which otherwise impact the leaf-to-air vapour gradient. The *g*_sw_ was calculated from energy balance equations for leaf temperature but, since latent heat flux from the non-measured leaf surface was not accounted for in this configuration, absolute *g*_sw_ values may contain systematic bias. Interpretation therefore focuses on relative temporal dynamics and treatment differences within each leaf side rather than absolute magnitude.

Leaves were first acclimated in the cuvette for 30–45 min at 0.7 kPa VPD_air_, matching the growth environment, and then exposed for 45 min to 1.35 kPa VPD_air_, then again for 45 min to 0.7 kPa. The resulting cuvette air conditions during the step changes can be found in Supplementary Fig. S6. Measurements were made on 3–4 plants per treatment for each replication, separately on the adaxial and abaxial sides following the manufacturer’s protocol (Li-Cor Biosciences, 2025), alternating which leaf side was used to start. The data were made continuous using monotonic cube spline interpolation (R splinefun, method=‘monoH.FC’). Each time series was visually checked to confirm that interpolated values followed the raw trend. Measurements from one plant were omitted owing to a technical issue with humidity regulation in the cuvette.

### Boundary layer measurement and calculations

The boundary layer conductance to water loss (*g*_bw_) was measured from the *E* obtained from wet filter paper by using Equation 1 (Jones, 2013).


(1) $$g_{\mathrm{bw}}=\;E/[\left(0.622\rho_{a}/P)(e_{s}-e_{a}\right)]$$

where ρ_a_ is the density of air, *P* is the atmospheric pressure, *e*_s_ is the saturated vapour pressure of the filter paper and *e*_a_ the vapour pressure of the air. Whatman No. 3 filter paper (GE Healthcare Life Sciences, Eindhoven, The Netherlands) was cut to the approximate shape, size, and proportions of a scanned lettuce leaf (32 cm^2^) with a characteristic dimension (L) of 5.38 cm. A black paper cutout was used as template for artificial leaves and was analysed using a custom Python script. The vertical distance between the upper and lower leaf edges was computed for every image column. For each column, the characteristic length was calculated, and the characteristic dimension was then obtained as the mean across all columns.

The filter paper was fully wetted and suspended horizontally by transparent fishing wire, which minimized interference with air movement. Evaporation was measured gravimetrically using the Fischer Scientific precision balance. Airflow next to the transpiring surface was measured with an NIST-calibrated hot-wire anemometer (TSI 8475, TSI Incorporated), positioned ∼2 cm from the filter paper. Air temperature and humidity were measured with a miniature humidity and temperature sensor (SEK-SHT40, Sensirion AG) and surface temperature with an infrared thermometer (Raynger ST, Raytek Corporation).

The total conductance to water vapour *g*_tw_ was calculated according to Equation 2.


(2) $$g_{\mathrm{tw}}=\frac{1}{\frac{1}{g_{b}}\;+\;\frac{1}{g_{s}}}$$

To quantify the influence of the atmosphere and the stomata on transpiration, the coupling factor was calculated, which helps to interpret the extent of stomatal control on transpiration (Jarvis and McNaughton, 1986) (Equation 3). The coupling factor (Ω) ranges between 0 and 1. When it is close to 0, *g*_b_ is high and transpiration is mainly controlled by *g*_s_, whereas if it is close to 1 the leaf is decoupled from the atmosphere and *g*_s_ has little impact on transpiration, leading to low stomatal control.


(3) $$\Omega =\frac{\varepsilon \;+\;1}{\varepsilon \;+\;1\;+\;\frac{g_{b}}{g_{s}}}$$

where ɛ is a temperature-dependent function defined by Jarvis and McNaughton (1986) (Equation 4) that measures how sensitive evaporation and transpiration are to temperature changes relative to their sensitivity to atmospheric conditions (Equation 4).


(4) $$\varepsilon =\frac{s\lambda }{C_{p}P}$$

where *s* is the slope of the saturation vapour pressure curve (kPa K^−1^), λ is the molar latent heat of vaporization of water (J mol^−1^), *C*_p_ is the molar heat capacity of air (J mol^−1^ K^−1^), and *P* is atmospheric pressure (kPa).

### Statistical setup and analysis

#### Butterhead lettuce

The experiment followed a randomized complete block design, with each experimental repetition serving as a block. Within each repetition, 28 lettuce plants were each exposed to two airflow conditions in the same climate chamber. To reduce positional bias, the fan positions were switched between repetitions, alternating the airflow+ and airflow– treatments. Plant responses were averaged within each treatment per repetition, resulting in a single paired value per treatment per repetition. The growth experiment and harvest were repeated six times (*n*=6). A subset of measurements (plant transpiration, leaf gas exchange, stomatal imprints, and microclimate) was performed on three to four repetitions (*n*=3–4) on the centre plants, which ensured that plants were measured and handled only once, and reduced mechanically induced responses. One additional supporting measurement (leaf cross-sections) was added only in the final two repetitions after initial results indicated their relevance, and was included to add complementary anatomical and physiological context.

Due to the block design, paired comparisons between treatments were generally performed on the averaged values using paired *t*-tests. When two fixed factors were analysed (e.g. treatment×leaf side or treatment×time), responses were first averaged per repetition×treatment×factor level, and then analysed with a linear mixed-effects model (Bates *et al*., 2015; Kuznetsova *et al*., 2017). The model specified treatment and the second factor (leaf side or time) as fixed effects, including their interaction term, while accounting for the block design by treating repetition as a random intercept. Mean comparison was then done with the emmeans package (Lenth, 2021). Residual normality and homoscedasticity were assessed using QQ and residuals-fitted plots. All statistical analyses were conducted in R (version 4.2.2) (R Development Core Team, 2021) using RStudio and the Tidyverse packages for data cleaning and plotting (Wickham *et al*., 2019).

#### Dwarf tomato

The dwarf tomato experiment similarly followed a randomized complete block design, with each of the 12 compartments serving as independent experimental units. The two central plants per compartment were measured and averaged, resulting in six replicates per airflow treatment (*n*=6). Compartments were evenly distributed across the left and right sides of the room, and this spatial division was included as a blocking factor. Because replicates were not paired, treatment effects were analysed using linear models with treatment and side of the room as fixed effects. Mean comparisons were obtained using estimated marginal means, and model assumptions were checked using residual diagnostics.

## Results

### Airflow-driven changes in leaf and canopy microclimate

#### Above-canopy microclimate

Although both treatments received the same incoming air, small but significant daytime differences in above-canopy air temperature and vapour pressure deficit (VPD_air_) were detected between airflow– and airflow+ treatments in the lettuce compartments (Supplementary Table S1). Daytime air temperature above the lettuce canopy was on average 0.44 °C lower and VPD_air_ 0.054 kPa lower under airflow+ compared with airflow– (*P*<0.05, *n*=6). No treatment differences were observed at night. Similarly, in dwarf tomato, small but significant shifts in the above-canopy microclimate were observed, although in this setup, VPD_air_ was slightly higher under airflow+ compared with airflow–, and no significant difference between air temperature was observed (Supplementary Table S2).

#### Canopy and leaf microclimate

**Fig. 1.**
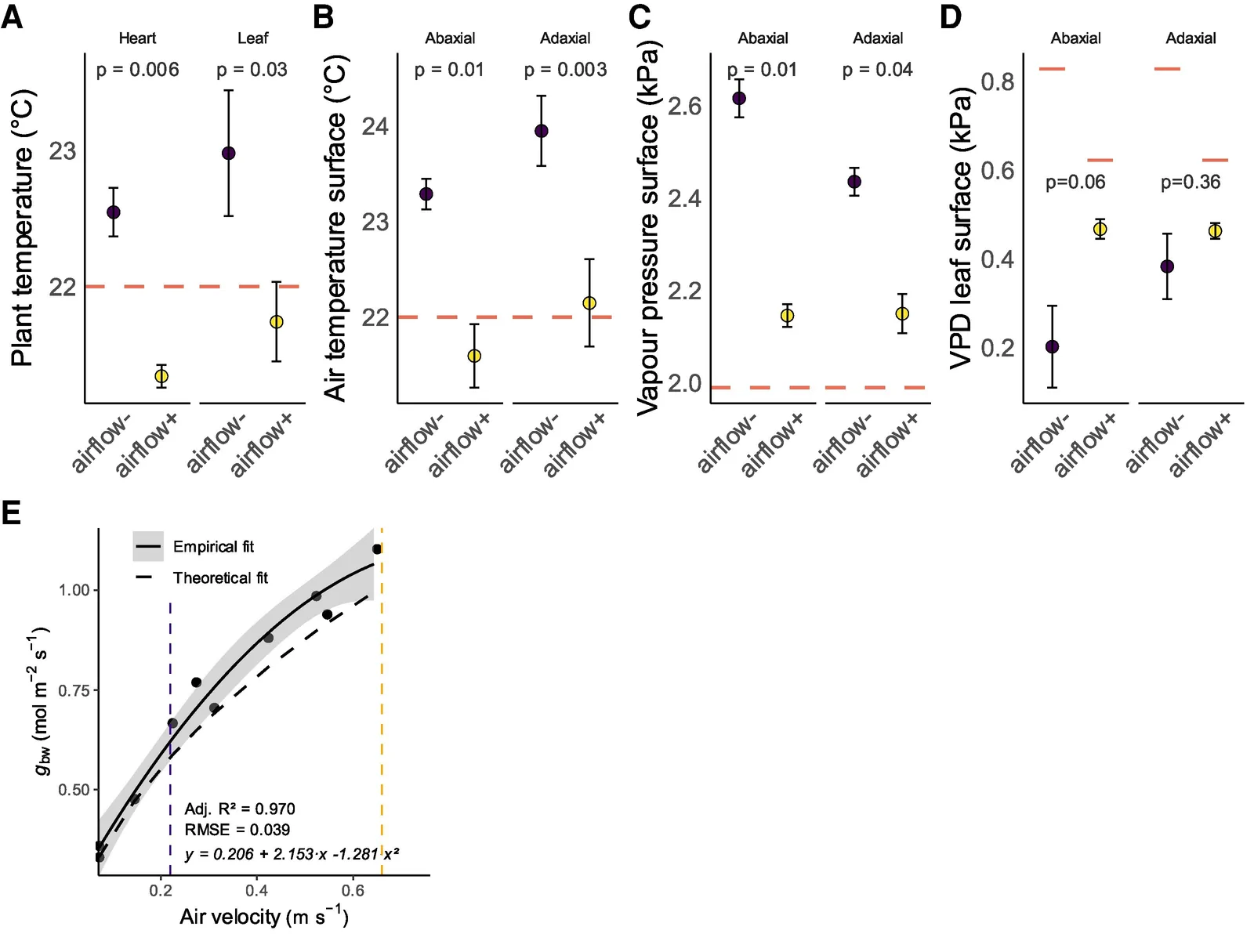
Leaf microclimate under low and enhanced airflow growth treatment (airflow– 0.22 m s^−1^ and airflow+ 0.65 m s^−1^). Horizontal red dashed lines mark the incoming air in the climate cell. (A) The plant temperature (°C) measured as leaf and heart temperature. (B–D) The air temperature (°C), the vapour pressure (kPa), and the vapour pressure deficit (VPD) at the abaxial and adaxial side of the leaf surface, calculated using the leaf temperature. Data are the mean ±SE. Mean values are treatment means of three growth cycles (*n*=3), each consisting of 14 averaged replicate plants for plant temperature and six for the side-specific measurement. *P*-values were obtained with a paired *t*-test. (E) Two-sided boundary layer conductance to water vapour (mol H_2_O m^−2^ s^−1^), with the theoretical fit following physical boundary layer theory for forced convection. Vertical dashed lines indicate the two treatments (airflow+ and airflow–).

Meristem temperature, approximated using the lettuce heart temperature, was 22.55±0.18 °C under airflow– conditions and decreased to 21.34±0.08 °C under airflow+ (Fig. 1A), which represented a consistent cooling effect (*P*=0.006). Leaf temperature followed a similar direction, averaging 22.98±0.45 °C under airflow– and 21.74±0.29 °C under airflow+ (*P*=0.03), with airflow– leaves slightly warmer and airflow+ leaves slightly cooler than the incoming air temperature setpoint (dashed line Fig. 1A). Air temperatures and vapour pressures measured adjacent to both the abaxial and adaxial leaf sides were also lower in airflow+ compared with airflow– (Fig. 1B, C, *P*<0.05). The VPD_surface_, calculated using the average leaf temperatures, showed a trend on the abaxial side, where VPD_surface_ was higher under airflow+ compared with airflow– (0.27 kPa, *P*=0.06), while on the adaxial side this pattern was not evident (Fig. 1D, *P*=0.36). Boundary layer conductance to water vapour (*g*_bw_) increased with airflow velocity (Fig. 1E), from 0.62 mol m^−2^ s^−1^ at 0.22 m s^−1^ to 1.06 mol m^−2^ s^−1^ at 0.65 m s^−1^. Despite identical incoming air and growth conditions, increased convective and diffusive exchange drove differences in leaf and canopy microclimate, leading to both the lettuce leaf and meristem experiencing a significantly cooler and less humid immediate environment under airflow+ (*P*<0.05).

### Plant growth and development

#### Growth parameters after 4 weeks of exposure to low airflow

After 4 weeks of growth, all plant morphology traits with the exception of SLA were significantly reduced (*P*<0.05) in lettuce plants exposed to enhanced airflow (airflow+) (Table 1). The plants displayed a significant reduction in growth, with fresh weight declining by nearly 13% compared with the low-airflow control. This loss of biomass was largely explained by an 8.6% decrease in leaf area, itself driven at least partially by a 7% decrease in leaf number, while SLA remained unchanged. Dry weight was reduced by 6%, and tissue water content (TWC) was similar (∼96%). Hence, the reduction in dry weight was proportional to the reduction in leaf area, while SLA and TWC were unchanged. Thus, differences between treatments reflected changes in total leaf area rather than changes in leaf mass per area. Tip burn, a major quality defect, was almost eliminated under the airflow+ treatment, which fell from >70% of plants to <1%. At the leaf level, the stomatal density and ratio remained unchanged between treatments. Because daytime air temperature under airflow+ was slightly lower, accumulated thermal time (heat units) over the growth period was calculated to assess potential temperature-driven effects. Thermal time differed by 2% when calculated from air temperature (*P*=0.022) (Supplementary Table S1). However, when the daytime heart temperature was used instead of air temperature, while retaining air temperature for the night period, the estimated difference in accumulated thermal time increased to ∼6.5% (Supplementary Table S3).

**Table 1.** Summary statistics of growth and development parameters after destructive harvest of lettuce plants grown for 28 d under 0.22 m s^−1^ (airflow–) and 0.65 m s^−1^ (airflow+)

	Airflow–	Airflow+	Difference (%)	Difference (absolute)	Diff. 95% CI	*P*-value
Whole-plant morphology						
Fresh weight (g)	49.13±4.2	42.82±3.4	−12.84%	6.31±1.08	3.53, −9.08	0.002
Leaf area (cm^2^)	906.1±58.6	828.1±52.5	−8.61%	78.05±22.0	21.5, 135	0.02
Dry weight (g)	1.80±0.13	1.69±0.11	−6.11%	0.11±0.03	0.04, 0.18	0.01
Leaves (no. per plant)	27.94±1.6	25.99±1.2	−6.98%	1.95±0.42	0.88, 3.02	0.005
SLA (cm^2^ g^−1^)	508.72±6.3	493.4±11.7	−3.01%	15.32±8.7	−7.09, 37.7	0.14
TWC (%)	96.25%	95.95%	−0.30%	0.0029±0.0003	n.a.	n.a.
Tip burn (%)	74.37%	0.66%	−99.04%	73.70±7.81	n.a.	n.a.
Leaf morphology						
Stomatal density adaxial (no. mm^−2^)	70.0±0.74	69.3±2.96	−0.97%	0.68±3.5	−15.8, 14.4	0.86
Stomatal density abaxial (no. mm^−2^)	47.4±1.92	45.1±1.45	−4.83%	2.29±3.32	−16.6, 12	0.56
Stomatal ratio	1.52±0.04	1.58±0.07	+3.95%	0.06±0.09	−0.35, 0.47	0.59

Data are the mean ±SE or the mean difference ±the SE of the mean difference, unless otherwise specified. Mean values are treatment means of six growth cycles for the whole-plant morphology measurements (*n*=6), each consisting of 20–24 averaged replicate plants, and three growth cycles (*n*=3) for the leaf morphology measurements, each consisting of four averaged replicate plants. The CI is the 95% confidence interval for the mean difference. *P*-values were obtained with a paired *t*-test. TWC, tissue water content; SLA, specific leaf area.

#### Growth dynamic

**Fig. 2.**
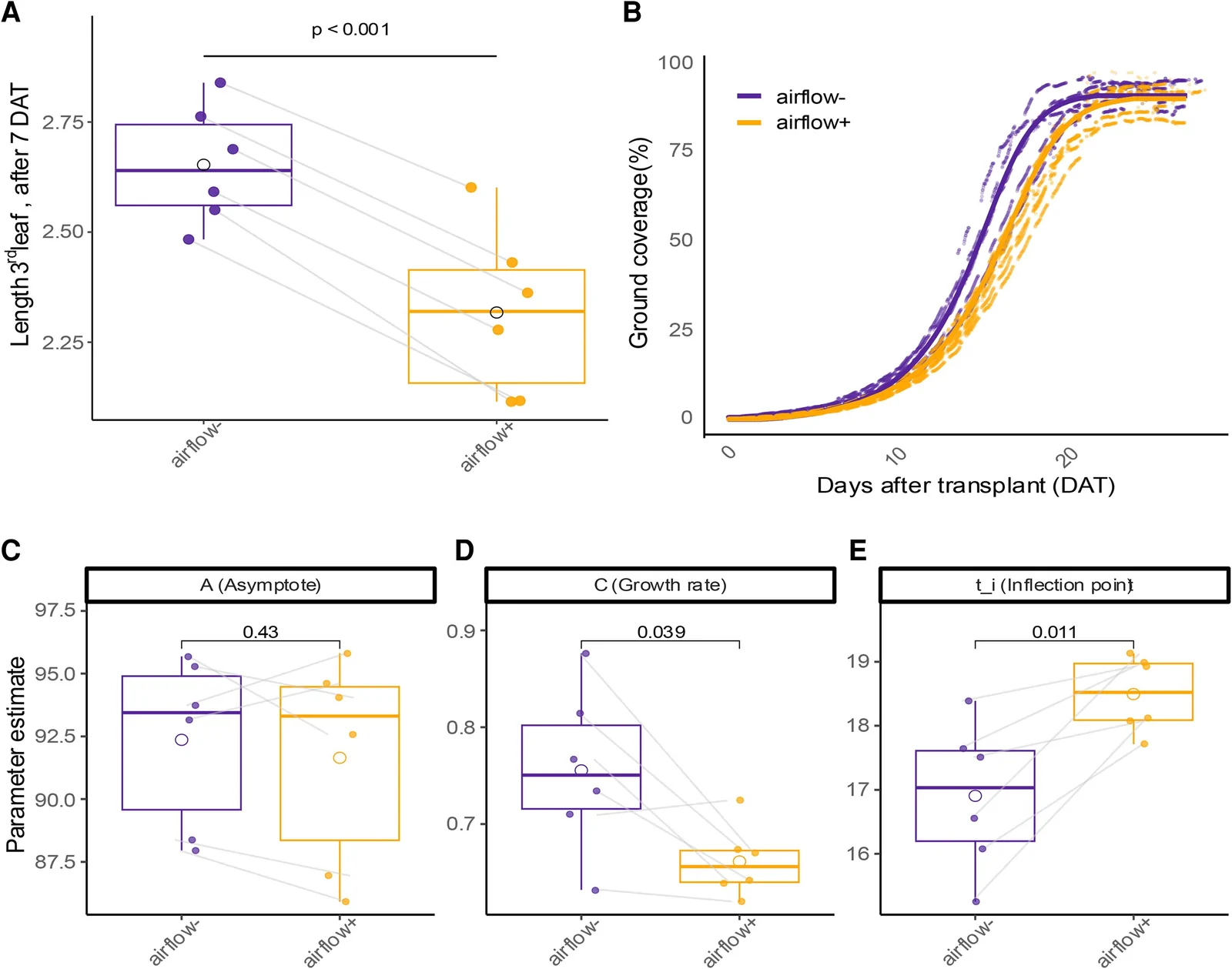
Leaf length and plant ground coverage response to low and enhanced airflow growth treatment (airflow– 0.22 m s^−1^ and airflow+ 0.65 m s^−1^). (A) The length of the third leaf, measured 7 days after transplant (DAT). This was the first leaf to emerge under the airflow treatments, and followed the cotyledons. (B) Ground coverage evolution after transplant until harvest after 4 weeks of growth. Circles indicate raw data from each growth cycle, and the fitted line represents the logistic curve obtained using average parameter values across cycles. (C) Parameters of the logistic function fitted to the ground coverage data: A (asymptote), C (growth rate), and t_i (inflection point). Boxplots display the median; mean values are indicated by open circles, and filled circles represent the individual replicates. Reported mean values are treatment means across six growth cycles (*n*=6); each cycle comprised 20–24 averaged replicate plants for ground coverage and 14 averaged plants for leaf length.

The first lettuce leaf formed under the treatment (third leaf overall) was shorter under airflow+, with the length significantly reduced by 12.6% (*P*<0.001) compared with the control (Fig. 2A). Ground coverage followed a typical logistic growth pattern in both treatments, with a rapid expansion phase followed by a plateau towards canopy closure (Fig. 2B). Despite the fact that both treatments ultimately reached a similar final coverage (Fig. 2C, *P*=0.43), airflow+ plants had a lower canopy growth rate (Fig. 2D, *P*=0.039), and required more time to reach their maximum growth rate, reflected in a delayed inflection point (Fig. 2E, *P*=0.011). These results demonstrated that the overall progression of canopy establishment, through both leaf initiation and expansion, was slowed under enhanced airflow. Consistent with these findings, a follow-up experiment with dwarf tomato showed comparable reductions in early vegetative growth (Supplementary Fig. S7) and fruit development under airflow+ (Supplementary Table S4).

### Gas exchange

#### Whole-plant gas exchange

**Fig. 3.**
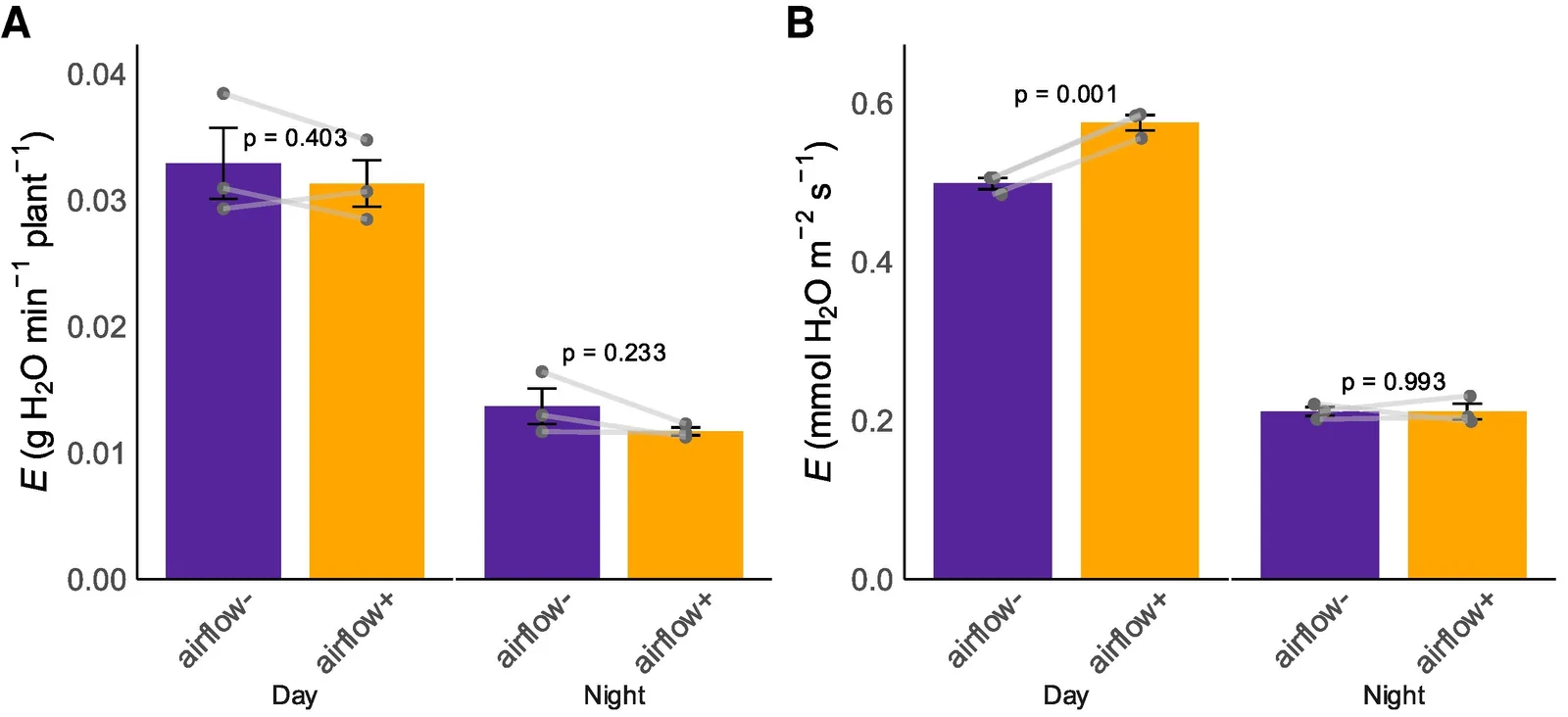
Whole-plant transpiration under low and enhanced airflow growth treatments (airflow–0.22 m s^−1^ and airflow+ 0.65 m s^−1^), measured gravimetrically in the growth environment. (A) Total whole-plant transpiration rate, expressed per plant. (B) Whole-plant transpiration rate, expressed by dividing the transpiration rate by the total leaf area of the same plants, measured immediately after gravimetrical measurements. Bars display treatment means, with errors bars representing the SE of three replications (*n*=3), which comprised three averaged plants each. Grey dots and connecting lines show paired values for individual growth cycles. *P*-values were obtained by paired *t*-tests.

Whole-plant transpiration (*E*) was measured gravimetrically under the respective airflow treatments in the growth environment, with the plant taken out from the canopy. Transpiration remained constant over time, as reflected by a linear decrease in plant weight during both the photoperiod and night, but with a steeper slope during the day than at night, which reflected a higher *E* during the photoperiod (Supplementary Fig. S8; summarized in Fig. 3). Total whole-plant *E* was similar between treatments, with no apparent differences in total water loss per plant during the photoperiod and at night (Fig. 3A, *P*=0.403 and 0.233). However, when whole-plant *E* was expressed per unit leaf area, *E* per unit leaf area was higher under enhanced airflow, but only during the daytime (15.2%, *P*=0.001) and not at night (*P*=0.993) (Fig. 3B).

#### Leaf gas exchange: stomatal behaviour under sustained airflow

**Fig. 4.**
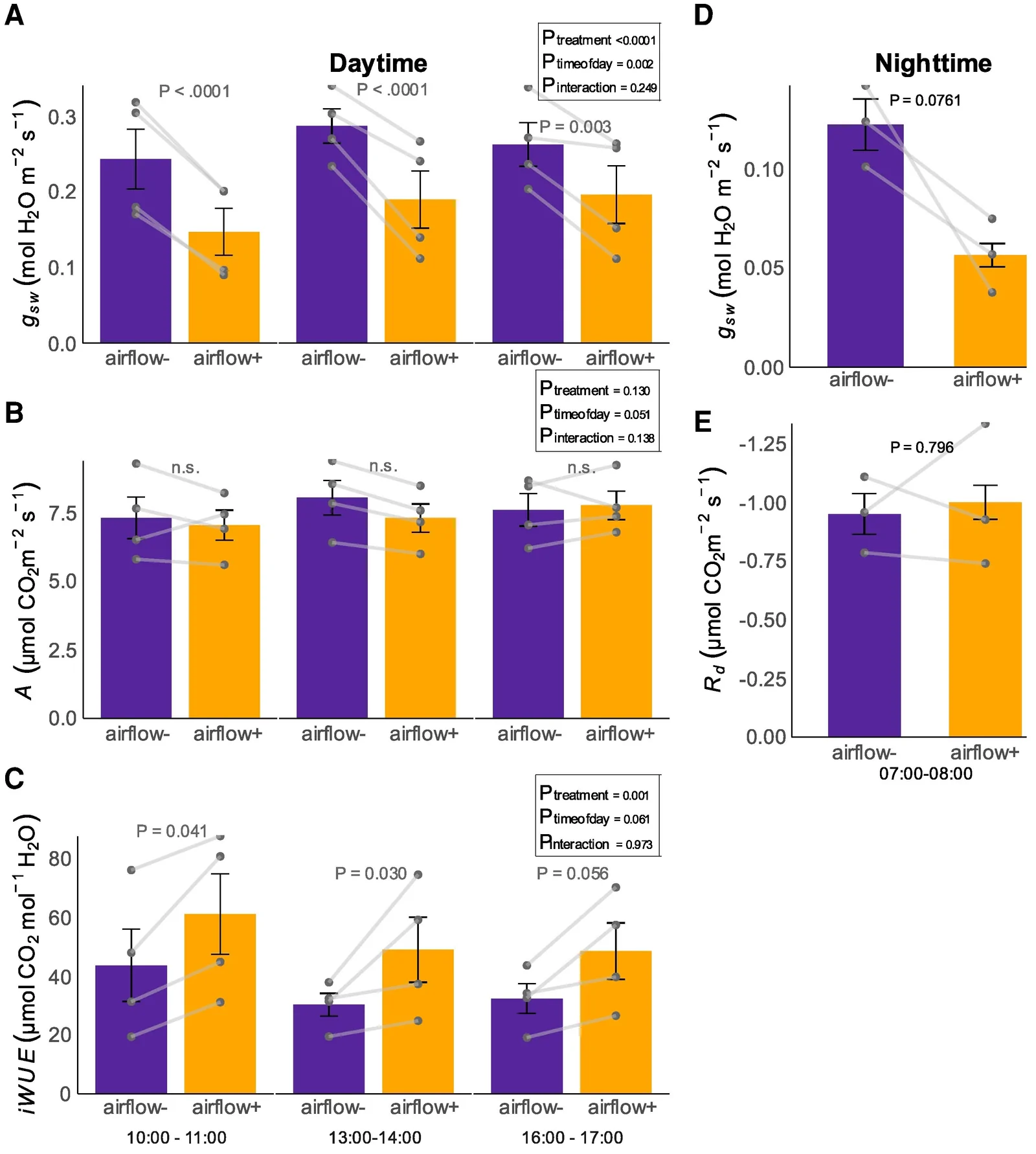
Leaf-level gas exchange snapshots taken during the daytime and night-time under low and enhanced airflow growth treatments (airflow– 0.22 m s^−1^ and airflow+ 0.65 m s^−1^) with a portable gas exchange system. (A) Daytime stomatal conductance (*g*_sw_). (B) Daytime net photosynthetic rate (*A*). (C) Daytime intrinsic water use efficiency (iWUE), calculated as the ratio of *A* to *g*_sw_. (D) Night-time *g*_sw_. (E) Night-time respiration rate (*R*_d_). Bars display treatment means, with errors bars representing the SE of 3–4 growth cycles (*n*=3–4), which consisted of six averaged replicate plants each. Grey dots and lines represent the paired growth cycles. *P*-values for the daytime measurements were obtained by pairwise comparisons only if the mixed linear model indicated a fixed treatment effect using a 0.05 cut-off. For the night-time measurements, *P*-values were obtained by paired *t*-tests.

To further explore whether leaf-level stomatal regulation limited an even further rise in *E* under airflow+, we conducted leaf-level gas exchange snapshots. This allowed us to assess whether changes in *g*_sw_ might have partially counteracted the potentially large increase in total conductance to water vapour (*g*_tw_), resulting from the higher *g*_bw_. During the photoperiod, airflow+ decreased *g*_sw_ compared with airflow– (*P*<0.001), and this decrease was consistent regardless of the time of the day (*P*-value interaction=0.25), and ranged from 25% to 39% (Fig. 4A). Although plants were grown under different airflow regimes, net photosynthetic rates (*A*) quickly equilibrated to similar values under identical cuvette reference conditions (Fig. 4B) (*P*=0.13). Thus, when measured under high *g*_bw_ conditions in a portable gas exchange system, the decrease in *g*_sw_ did not limit photosynthesis. Any difference in *A* under similar conditions would not represent the operational conditions in the growth environment. The decrease in *g*_sw_ paired with no observable impact on *A* led to a higher iWUE under airflow+ (*P*=0.001), with increases between 40% and 62% (Fig. 4C). During the nocturnal period, a similar trend was observed (Fig. 4D), with nocturnal *g*_sw_ decreased by 50% under airflow+ compared with airflow– (*P*=0.076). Night-time respiration did not significantly differ between treatments (Fig. 4E). The follow-up experiment with dwarf tomato similarly displayed significant reductions of 46% in *g*_sw_ under airflow+, accompanied by a 16% reduction in *A* and a 42% increase in iWUE (Supplementary Fig. S9). Because iWUE was calculated with *A* values that quickly equilibrate to the cuvette conditions, it was mainly determined by the slow stomatal component of airflow responses and excludes potential diffusive limitations by low *g*_b_ during measurement.

#### Leaf-to-air coupling: relative contribution of stomatal and boundary layer conductance in gas exchange

To assess the combined impact of the stomatal response (*g*_sw_) and boundary layer (*g*_bw_) and to determine which exerted the dominant control over transpiration and WUE under different airflow conditions, the total conductance to water vapour (*g*_tw_) and the coupling factor (Ω) were calculated under the two airflow treatments (Table 2). Despite the strong increase in *g*_bw_ under airflow+ (+72.3%), this was effectively counterbalanced by a reduction in *g*_sw_ (−32.8%), such that *g*_tw_ showed a declining trend (−18.2%, *P*=0.067). This tighter control of *g*_sw_ is more clearly reflected in Ω, which integrates the relative influence of *g*_bw_ and *g*_sw_ on transpiration. Values approaching 0 indicate strong coupling (stomata exert greater control), while values near 1 indicate weak coupling (the environment dominates transpiration). The Ω decreased from 0.586 to 0.349 under airflow+ (−40.4%, *P*<0.001), which indicated tighter coupling of leaves to the atmosphere and stronger stomatal regulation of transpiration. The relative contribution of *g*_bw_ to *g*_tw_ decreased under enhanced airflow, as stomatal diffusive limitation increasingly dominated *g*_tw_.

**Table 2.** Effects of low and enhanced airflow growth treatments (airflow– 0.22 m s^−1^ and airflow+: 0.65 m s^−1^) on the total conductance to water vapour and the leaf–atmosphere coupling

	*g*_sw_	*g*_bw_	*g*_tw_	Ω
Airflow–	0.264±0.03	0.62	0.181±0.01	0.586±0.03
Airflow+	0.178±0.04	1.06	0.148±0.03	0.349±0.05
Diff. (%)	−32.8%	+72.3%	−18.2%	−40.4%
Diff.	0.087±0.01	0.446	0.033±0.01	0.223±0.01
95% CI	0.057–0.116	n.a.	0–0.070	0.185–0.261
*P*-value	0.003	n.a.	0.067	<0.001

For stomatal conductance, mean values per day from Fig. 4 were used. *g*_sw_, stomatal conductance; *g*_bw_, boundary layer conductance; *g*_tw_, total conductance to water vapour; Ω, coupling factor. When Ω approaches 0, *g*_b_ is high and transpiration is primarily controlled by *g*_s_, whereas values close to 1 indicate strong decoupling of the leaf from the atmosphere, and the environment (e.g. radiation) determines transpiration. The CI is the 95% confidence interval for the mean difference. *P*-values were obtained by paired *t*-tests (*n*=4).

To further assess how *g*_b_ in the growth environment influences CO_2_ supply and WUE, a theoretical photosynthetic rate (*A*_theory_) was recalculated. Fick’s diffusion equation was applied using the growth environment *g*_b_, replacing the artificially elevated *g*_b_ imposed by the gas exchange cuvette. As these values are theoretical outputs derived from measured parameters, differences are presented descriptively. For each leaf, it was assumed that the ratio *C*_i_/*C*_a_, and thus the drawdown imposed by leaf biochemical demand, remained unchanged.

Under cuvette conditions, measured *A* was 7.67 μmol CO_2_ m^−2^ s^−1^ in airflow– and 7.39 μmol CO_2_ m^−2^ s^−1^ in airflow+ (previously presented in Fig. 4B). When recalculated using *g*_b_ values characteristic of the growth environment, *A*_theory_ was 6.33 μmol CO_2_ m^−2^ s^−1^ in airflow– and 7.00 μmol CO_2_ m^−2^ s^−1^ in airflow+, corresponding to a 10.47% increase under airflow+. Similarly, we recalculated a theoretical transpiration rate (*E*_theory_leaf_). Under cuvette conditions, measured *E* averaged 1.45 mmol H_2_O m^−2^ s^−1^ in airflow– and 1.01 mmol H_2_O m^−2^ s^−1^ in airflow+. When recalculated with environmental *g*_b_, *E*_theory_leaf_ was 1.02 mmol H_2_O m^−2^ s^−1^ in airflow– and 0.85 mmol H_2_O m^−2^ s^−1^ in airflow+. Consequently, theoretical instantaneous water use efficiency (instWUT_theory_) averaged 7.07 μmol CO_2_ mmol^−1^ H_2_O in airflow– and 9.70 μmol CO_2_ mmol^−1^ H_2_O in airflow+, representing an ∼37% increase under enhanced airflow. The magnitude was similar to that observed in iWUE under cuvette conditions. This pattern is consistent with treatment effects being largely mediated through changes in *g*_s_ rather than through the direct physical influence of *g*_b_ alone, although this simplified reconstruction does not allow us to fully resolve these interactions. These reconstructions were performed under standardized cuvette conditions that maintained a constant driving force (VPD_leaf_ and CO_2_ concentrations). Under cuvette conditions, strong leaf–air coupling minimizes boundary-layer-induced gradients, tightly linking stomatal responses to the imposed atmosphere. In contrast, lower *g*_b_ under the growth environment, weakens this coupling, allowing the leaf surface microclimate to diverge from ambient conditions (Fig. 1). The impact of these microclimate differences that occur in the growth environment were therefore not fully captured.

#### Theoretical influence of leaf surface microclimate on plant transpiration

Using the measured leaf conductances and vapour gradients, a theoretical plant transpiration rate (*E*_theory_plant_) was calculated to compare with the measured whole-plant transpiration. When using the leaf-to-air vapour gradient measured above the canopy, *E*_theory_plant_ was significantly higher for airflow– (1.28 mmol H_2_O m^−2^ s^−1^) than airflow+ (0.79 mmol H_2_O m^−2^ s^−1^). This was unexpected and was the opposite response compared with the measured values, where *E*_plant_ per leaf area was higher for airflow+ (Fig. 3B). However, using vapour gradients measured near the leaf surface (Fig. 1C, assuming both leaf sides contribute similarly) in the *E*_theory_plant_ calculation produced values for airflow– (0.53 mmol H_2_O m^−2^ s^−1^) and airflow+ (0.70 mmol H_2_O m^−2^ s^−1^) that were more aligned with the measured whole-plant *E*.

#### Leaf side-specific gas exchange responsiveness to vapour presure deficit

**Fig. 5.**
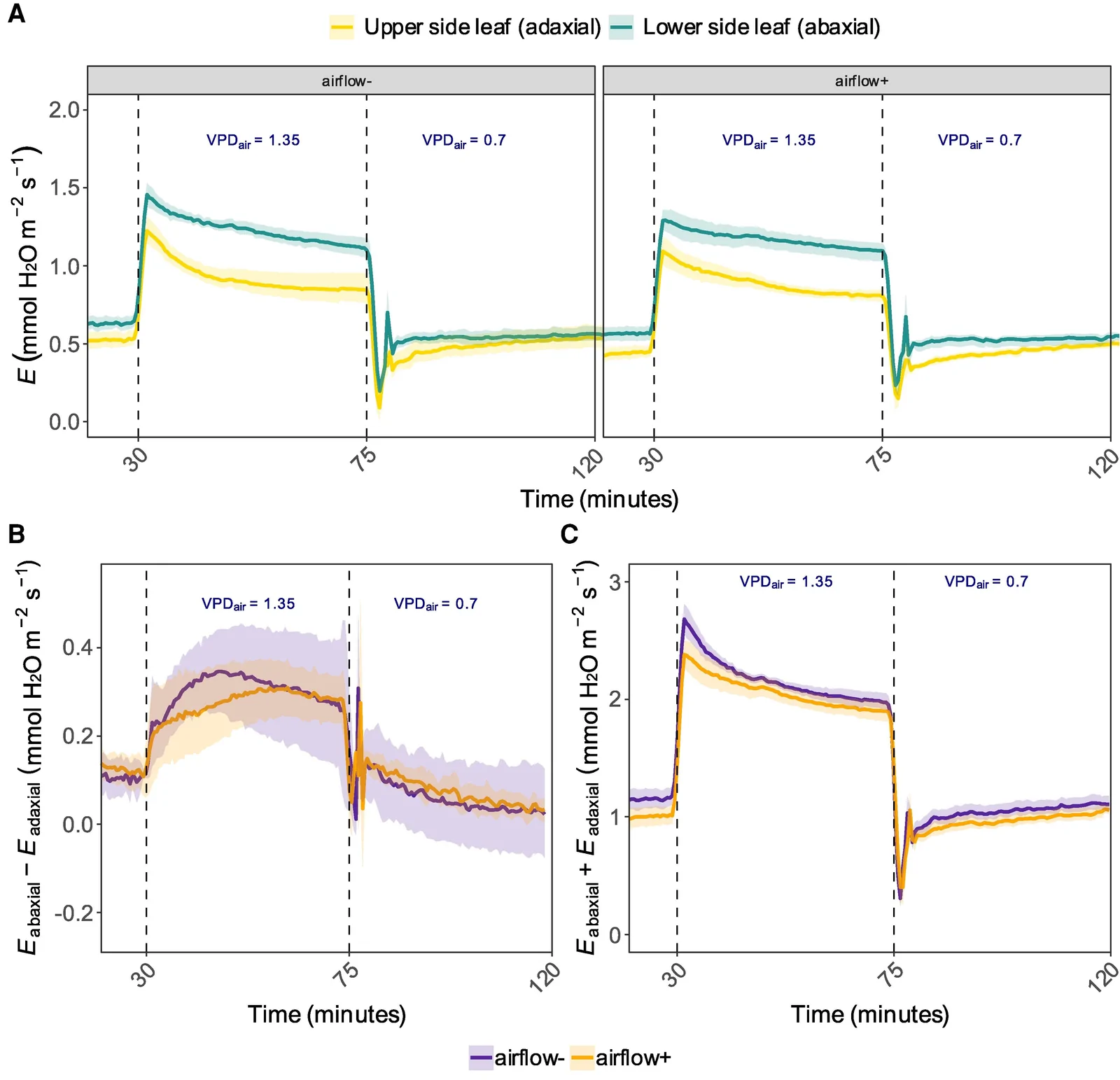
Leaf transpiration responses of adaxial and abaxial leaf sides of plants grown under low and enhanced airflow growth treatments (airflow– 0.22 m s^−1^ and airflow+ 0.65 m s^−1^ to step changes in VPD_air_. (A) Transpiration rates (*E*) of the abaxial and adaxial leaf sides. (B) The difference between abaxial and adaxial leaf side. (C) Sum of the abaxial and adaxial leaf side. Mean values are treatment means of three growth cycles (*n*=3), which each consisted of 3–4 averaged replicate plants per treatment.

To assess whether prior airflow exposure altered stomatal responsiveness (i.e. evidence of acclimation) and to examine side-specific sensitivity to diffusive demand, abaxial and adaxial responses to a step change in VPD_air_ were measured, as alterations in VPD_air_ could be one of the driving forces of altered stomatal behaviour under airflow+. Because the cuvette environment was controlled, changes in *E* primarily reflected changes in *g*_sw_ and minimally changes in leaf temperature. Following an increase in VPD_air_, *E* rose sharply and almost instantaneously, before gradually declining in both the adaxial and abaxial sides (Fig. 5A). To compare treatments, the difference (Δ) in *E* was calculated as the change between the maximum and minimum values observed during the VPD_air_ step change (Δ*_E_*=*E*_max_−*E*_min_). Both airflow+ and airflow– plants gradually decreased their transpiration in response to a lower humidity (Table 3), after the initial rise. The decrease in *E* was affected by the treatment (*P*=0.019) and was lower in airflow+, but post-hoc pairwise comparisons within each leaf side showed only a trend towards a weaker decrease in airflow+ plants (*P* ∼0.06–0.07). This slight discrepancy probably reflected the lower statistical power of the within-side contrasts, as the main treatment effect was consistent across both leaf sides and no significant interaction was detected (*P*=0.90). Leaf side effects were not significant and the contribution of the abaxial versus adaxial side differed little between treatments (Fig. 5B). Thus, the relative contribution of each leaf side to total *E* changed similarly in both sides after the VPD_air_ step, but the overall magnitude of the response differed between airflow treatments. Despite this, the total *E* (i.e. summing both leaf sides) stabilized to similar values among treatments towards the end of the step change (Fig. 5C).

**Table 3.** Summary of leaf transpiration response (Δ*_E_*=*E*_max_−*E*_min_) and net photosynthetic rate (Δ*_A_*=*A*_time30_−*A*_time75_) to a VPD step change, comparing two growth treatments (airflow– 0.22 m s^−1^ and airflow+ 0.65 m s^−1^) across the abaxial and adaxial leaf sides

	Airflow–	Airflow+	
*E*	Δ*_E_*=*E*_max_−*E*_min_	Δ*_E_*=*E*_max_−*E*_min_	*P*-value treatment
Abaxial	−0.344±0.054	−0.309±0.055	0.070
Adaxial	−0.417±0.013	−0.244±0.02	0.057
*P*-value leaf side	0.161	0.210	
*A*	Δ*_A_*=*A*_time30_−*A*_time70_	Δ*_A_*=*A*_time30_−*A*_time70_	*P*-value treatment
Abaxial	0.206±0.120	0.096±0.151	0.379
Adaxial	−0.427±0.073	−0.261±0.082	0.202
*P*-value leaf side	0.002	0.021	

*P*-values were obtained by pairwise comparisons after fitting a mixed linear model (*n*=3).

**Fig. 6.**
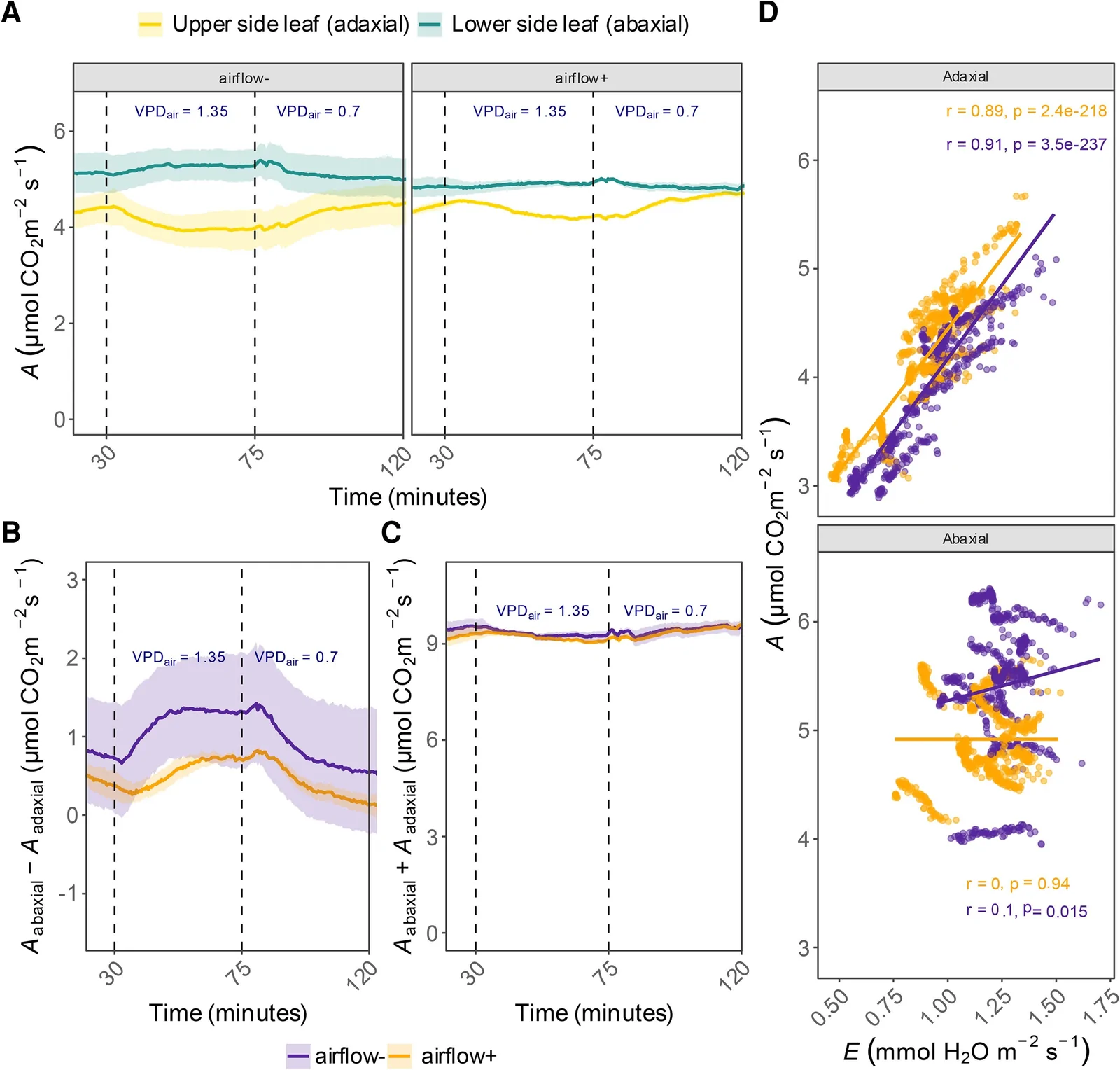
Leaf photosynthesis responses of adaxial and abaxial leaf sides to step changes in VPD_air_. (A) Net photosynthetic rates (*A*) of the abaxial and adaxial leaf sides. (B) The difference between the abaxial and adaxial leaf surface. (C) Sum of the abaxial and adaxial leaf side. (D) Relationship between *A* and *E* measured on adaxial and abaxial leaf sides during the 1.35 kPa VPD_air_ step. Mean values are treatment means of three growth cycles (*n*=3), each consisting of 3–4 averaged replicate plants per treatment.

Simultaneous measurements of *A* revealed that the VPD_air_ step change impacted the two leaf sides differently (Table 3; Fig. 6). For the adaxial side, *A* declined in response to higher VPD_air_, whereas the abaxial side remained relatively steady or even increased (Fig. 6A). Therefore, the Δ*_A_* was calculated as the difference between the value at time 30 and the value at time 70, to account for both declines and increases in *A*. The Δ*_A_* was larger on the adaxial side compared with the abaxial side (*P*<0.001), regardless of the airflow treatment (*P*-value interaction=0.14). There was no clear treatment effect (*P*=0.74), indicating that the reduction in *A* was primarily driven by leaf side rather than airflow treatment, which caused a clear shift in relative contribution of the leaf sides in response to VPD_air_ (Fig. 6B). Despite these shifts in relative side contribution, total leaf *A* remained constant, with no treatment differences observed (Fig. 6C).

We further examined the relationship between *A* and *E* to investigate photosynthetic limitation, and found that the relationship was significantly positive and strong on the adaxial side for both airflow– and airflow+ (Pearson’s *r* ∼0.90, *P*<0.001, Fig. 6D). In contrast, the abaxial sides showed only weak or no correlations among treatments (Pearson’s *r* <0.10). Overall, these results showed no treatment effect and that leaf gas exchange and diffusion were asymmetrical between leaf sides, with photosynthesis limited primarily on the adaxial side in response to the VPD_air_ step change. This limitation was linked to closure of stomata, as seen by the strong *A*/*E* correlation. Indeed, the trajectory of *g*_sw_ during the step change closely followed that of transpiration and was directionally similar on both leaf surfaces (Supplementary Fig. S10). However, absolute *g*_sw_ values were consistently lower on the adaxial side during the step change.

#### Internal leaf anatomy

To characterize anatomical differences between leaf sides, transverse leaf sections were analysed. Transverse leaf sections revealed a distinct anatomical asymmetry between the adaxial and abaxial sides (Supplementary Fig. S11). The adaxial side was characterized by densely packed palisade mesophyll cells with relatively little intercellular space, whereas the abaxial side exhibited a more open spongy mesophyll with extensive airspace between cells. Quantitative image analysis confirmed this visual pattern, and showed that airspace occupied a significantly greater proportion of tissue area on the abaxial side (45.25±0.13%) compared with the adaxial side (25.9±0.27%) (*P*=0.007) (Supplementary Fig. S11). No significant difference was observed across both treatments (*P*=0.11 and *P*-value interaction=0.40), demonstrating that the structural asymmetry was mainly an inherent feature of the different anatomy between leaf sides.

## Discussion

Acclimation to a modest increased airflow reduced lettuce fresh weight by 13% despite identical climate setpoints, driven by decreases in leaf initiation and expansion. This decrease was associated with the increased transpiration per unit leaf area under airflow+, for which stomatal closure did not fully compensate. Instead, stomata appeared to operate near a functional limit where further closure would have compromised photosynthesis (Figs 4, 6D; Supplementary Fig. S9), suggesting a trade-off between CO_2_ and H_2_O exchange, which shifts with airflow. These findings stress that subtle airflow differences in growth chambers affect short-term regulation and acclimation, ultimately impacting growth and yield (Table 1; Fig. 1).

### Small airflow velocity differences modified the leaf microclimate

Increased airflow produced slightly cooler leaves (>1 °C), lower surface humidity (>0.4 kPa), and steeper vapour gradients (Fig. 1), demonstrating that differences in airflow altered the microclimate of the plants. Although individually modest, these changes collectively strengthened leaf-to-air coupling and altered the plant’s energy balance (Table 2), consistent with previous reports of canopy microclimatic heterogeneity in CEA systems (Savvides *et al*., 2013; Kimura *et al*., 2020a, b; Plas *et al*., 2025), and modelling studies demonstrating that airflow modifies leaf surface microclimates (Collatz *et al*., 1991; Aphalo and Jarvis, 1993; Dupont *et al*., 2025). Importantly, the magnitude of this microclimatic modification depends on chamber design and operating conditions (Kang and van Hooff, 2024). In both growth chambers and gas exchange cuvettes, airflow (mainly determined by fan speed) interacts with inflow rate and climate control to reach environmental setpoints (VPD_air_, temperature, and CO_2_), such that low internal mixing, particularly when combined with a low inflow rate or strong pre-existing gradients, can amplify boundary layer thickening and local leaf microclimate deviations from bulk air conditions (Hupp and Vath, 2025). Airflow effects are therefore dependent on the room design, climate setpoints, and plant architecture that influence airflow distribution within the chamber.

Because airflow altered the leaf microclimate relative to bulk air conditions, the estimated transpiration response from diffusion theory depended strongly on how VPD_leaf_ was calculated. Using leaf-adjacent humidity, transpiration increased under airflow, whereas using above-canopy air humidity underestimated gradients. This emphasizes how unresolved within-canopy microclimates can mislead physiological interpretation (Savvides *et al*., 2013, 2017) and highlights an emerging property of low airflow environments: small microclimate perturbations accumulate at the leaf surface to drive unexpected plant responses.

### Dynamic regulation versus structural acclimation under airflow

Similar stomatal density, transpiration, and *g*_s_ were observed under reference conditions, with similar contribution of the abaxial versus adaxial side between treatments, which demonstrated that the stomatal response was rapidly reversible and not due to acclimation (Fig. 5; Supplementary Fig. S10; Table 1). Under sustained higher evaporative demand (e.g. VPD_air_ >2 kPa), dynamic stomatal regulation may reach its limits, requiring structural adjustments in stomatal or vein traits (Du *et al*., 2019), whereas some species maintain homeostasis using dynamic stomatal regulation alone (Carins Murphy *et al*., 2014). These contrasting strategies show that the shift from dynamic regulation to structural acclimation is species and hydraulics dependent (Tardieu and Simonneau, 1998; Fanourakis *et al*., 2016; Xiong and Nadal, 2020; Koehler *et al*., 2023). Our results therefore are likely to represent a conservative case in which subtle airflow differences primarily elicited dynamic regulation under otherwise mild growth chamber conditions (Fig. 1).

### Stomatal regulation partially compensated for increased diffusion under sustained airflow

Airflow typically triggers stomatal feedback to altered transpiration (Kuiper, 1961; Dixon and Grace, 1984; Bunce, 1985; Collatz *et al*., 1991; Aphalo and Jarvis, 1993; Schymanski and Or, 2016; Shapira *et al*., 2024), but most evidence stems from short-term studies (minutes to hours). Here, even minor differences in VPD_leaf_ (<0.27 kPa) induced strong stomatal responses (−33% in *g*_s_) under sustained airflow. Such responses are often overlooked in models assuming minimal stomatal sensitivity within this range (Grossiord *et al*., 2020), and probably reflect in whole-plant regulation of transpiration (Tardieu and Simonneau, 1998).

No substantial differences in *A* or *g*_s_ were observed under identical reference conditions (Fig. 6; Supplementary Fig. S10), suggesting an absence of acclimation, despite evidence that prolonged reductions in *g*_s_ (as observed here) or *g*_m_ can suppress photosynthetic capacity (Du *et al*., 2019; Grossiord *et al*., 2020). Instead, the higher leaf-level iWUE under enhanced airflow resulted from reversible reductions in *g*_s_, reflecting the operational regulations in response to airflow, supporting previous observations under short-term increased airflow and VPD_air_ (Schymanski and Or, 2016; Grossiord *et al*., 2020). Our calculations further indicate that *g*_b_ potentially played a role in diffusional limitation of *A*, impacting WUE. This effect is typically obscured in leaf cuvettes with artificially high *g*_b_, which can misrepresent leaf-level WUE.

Under fluctuating airflow, enhanced airflow may instead accelerate stomatal kinetics, increase stomatal opening, and transiently increase water loss (Shapira *et al*., 2024), probably through increased evaporative demand (Pichaco *et al*., 2024). However, most previous studies examined short-term airflow perturbations with single leaves in cuvettes. In contrast, our plants experienced sustained airflow during growth, and our snapshot measurements therefore reflected operational whole-plant regulation under steady airflow conditions (Tardieu and Simonneau, 1998; Anten *et al*., 2010). Under these conditions, slow stomatal regulation appeared to maintain transpiration within a sustainable range rather than amplify short-term increases in transpiration. Importantly, the effect of airflow on transpiration is likely to depend on the initial level of leaf evaporative demand. When transpiration is already high, stomatal regulation can limit further water loss, whereas at lower initial rates, airflow may enhance transpiration (Franks and Brodribb, 2005). Interactions among radiation load, *g*_s_ responses, and leaf energy balance can therefore explain why airflow sometimes increases or decreases transpiration (Schymanski and Or, 2016), potentially resolving some discrepancies in the literature. Furthermore, at the whole-plant scale, transpiration reflected the combined influence of *g*_s_, *g*_b_, and airflow-driven aerodynamic gradients (Figs 1, 3). Leaf- and plant-level responses were broadly similar, but interpretation at the whole-plant scale was even more complex due to spatial gradients that alter plant–climate coupling.

### Stomatal regulation imposes side-specific photosynthetic limitation

While stomata partially closed in response to enhanced airflow (Fig. 4), further closure began to restrict photosynthesis (Fig. 6; Supplementary Fig. S9), preventing further reduction in transpiration (Fig. 3). This suggests that the plant optimizes photosynthesis at the expense of transpiration in response to microclimatic variation (Cowan and Farquhar, 1977). Although not significant, the large reduction in nocturnal conductance (−50%) under high airflow is notable. Due to this strong closure, nocturnal transpiration remained unchanged, indicating that at night, when photosynthesis does not constrain stomatal regulation, stomata fully compensated for the increase in airflow. This pattern is consistent with the view that nocturnal conductance is a dynamically regulated, growth-linked trait that is down-regulated when its water cost rises (Resco de Dios *et al*., 2019; McAusland *et al*., 2021).

The photosynthetic limitation in response to VPD_air_ was side specific. Initially, we hypothesized that the adaxial side, more exposed to airflow, would have higher VPD_air_, lower *g*_s_, and more responsive stomata than the sheltered abaxial side. Such differences in VPD_air_ can increase stomatal responsiveness at the leaf level (Aliniaeifard *et al*., 2014; Carvalho *et al*., 2015), and therefore are expected to depend on airflow. However, stomatal kinetics differed only marginally between treatments while photosynthetic limitation differed only between leaf sides (Table 3). The adaxial side displayed reduced CO_2_ assimilation, which was linked to *g*_s_ decline and was similar among treatments (Fig. 6D). The adaxial side had less intercellular airspace (26% versus 45%), and higher stomatal density, but lower initial *g*_s_ values (Table 1; Fig. 6; Supplementary Figs S10, S11). These features, together with higher irradiance in the upper mesophyll (Vogelmann and Evans, 2002; Evans *et al*., 2017), probably increased CO_2_ demand in the dense palisade cell layer and triggered an earlier photosynthetic limitation (Morison and Lawson, 2007). While differences in photosynthetic capacity and stomatal density between leaf sides are often cited as the primary explanation for asymmetrical responses (Fanourakis *et al*., 2015; Wall *et al*., 2022), our results suggest that differences in external diffusive gradients may also contribute, reinforcing that each leaf side operates at least partially independently.

### Airflow alters growth via temperature and hydraulic pathways

Our findings support a general hydraulic framework in which environmental cues that alter daytime and night-time transpiration modify expansive growth, consistent with evidence that leaf elongation responds to stomatal control of transpiration (Barillot *et al*., 2021). In that framework, changes in *g*_s_ (e.g. due to blue light or VPD_leaf_) rapidly modified transpiration, which shifted water potential gradients in the leaf growth zone, and thereby modulated elongation rates. Our results suggested that airflow acts through this same causal chain. Due to altered VPD_leaf_ and *g*_b_, increased transpiration probably imposed hydraulic limitations on expansive growth, which is sensitive to both cell turgor and water fluxes (Pantin *et al*., 2011, 2012), and subsequently reduced leaf elongation rates.

In lettuce, reduced plant temperature contributed to reduced leaf area (Fig. 1), as the 7% reduction in leaf number closely matched the 6.5% lower accumulated thermal time under airflow+ (Supplementary Table S3). Small meristem temperature deviations of just 1–2 °C at ∼20 °C substantially alter organogenesis (Savvides *et al*., 2016; Heuvelink, 2018). However, the reduction in biomass (13%) and the similar growth reductions in tomato, where airflow slightly warmed leaves (Supplementary Tables S2, S4; Supplementary Fig. S7), indicated that temperature alone cannot explain the faster canopy closure. Additionally, ground coverage trajectories indicated both a temporal shift (inflection point) that could be explained by thermal time, and also a reduction in growth rate (slope) (Fig. 2B). Instead, airflow-induced increases in transpiration probably imposed hydraulic constraints on leaf expansion or other strategies that reduce the effective leaf area (Renard and Demessemacker, 1983; Anten *et al*., 2010; Kadioglu *et al*., 2012). In addition to altered elongation, airflow+ showed a marked decrease in tip burn, which is commonly observed in lettuce (Frantz *et al*., 2004). An increased transpiration when the lettuce heart was formed may increase calcium transport and alleviate tip burn, at the cost of reduced leaf elongation at an early growth stage.

### When does airflow benefit or constrain the plant?

Airflow effects depend on how it shifts the transport balance of CO_2_, heat, and water, and by any concurrent mechanical stress (Grace, 1988; Anten *et al*., 2010; Dupont *et al*., 2025), which reconciles previous inconsistencies regarding its effect on growth. Modelling work under high light intensity predicted positive effects on leaf-level photosynthesis, especially under CO_2_ limitation or under thermal relief (Collatz *et al*., 1991; Ono *et al*., 2024, Preprint; Dupont *et al*., 2025), conditions that were largely absent in our experiment. However, under moderate light intensity like here, the reduction in expansive growth and leaf initiation was likely to be more important than the estimated 10% increase in leaf photosynthesis under increased airflow. Thus, in this experiment under moderate light, the dominant limitation on growth was probably not photosynthesis but light capture, which depends on factors such as water balance, developmental stage, and planting density (Frantz *et al*., 2004; Tardieu *et al*., 2015; Xiong and Nadal, 2020).

### Reporting airflow is essential for reproducible plant science

Our study stressed that ignoring airflow can lead to false attribution of plant responses to nominal chamber setpoints. A recent review emphasized that airflow is rarely documented (8 of 221 studies in Vincent *et al*., 2025), and here we emphasize that airflow requires standardized reporting. For reproducibility in growth chambers, we reiterate the recommendation of Both *et al*. (2015) to report the air circulation system design, resulting airflow velocity, and direction (including sensor type and placement). Importantly, airflow around leaves is not determined solely by chamber settings but is modified by canopy architecture and planting density. Estimating *g*_b_ provides an even more meaningful metric than airflow alone because it captures the non-linear velocity–conductance relationship, and incorporates the impact of turbulence and flow angle, and leaf shape. In our study, increased airflow altered important traits which are often interpreted without considering *g*_b_. This issue is especially relevant for high-throughput phenotyping, where small airflow differences between facilities, or between growth chambers and high-airflow cuvettes, could alter conclusions about WUE or stomatal control. Practical estimates of *g*_b_ (e.g. filter paper evaporation) may improve comparability across facilities.

In summary, we invalidate the tacit assumption that low airflow variations have minimal impact on plant acclimation. Acclimation to the altered microclimate occurred through decreased leaf initiation and leaf elongation, which was caused by an increase in transpiration despite a 33% reduction in *g*_s_. We propose that further stomatal closure was limited by the functional trade-off between water and carbon in response to mild microclimatic variations, and further water savings would have maintained leaf expansion to the detriment of carbon assimilation. As a consequence, increased airflow reduced yield by 13%, demonstrating that airflow can be as influential as other commonly researched climate variables.

## Supplementary Material

erag185_Supplementary_Data

## Data Availability

The primary data supporting this study were not made publicly available at the time of publication. Data will be made available upon request to the corresponding author (Silvere Vialet-Chabrand).

## Supplementary data

The following supplementary data are available at *JXB* online.

Fig. S1. Arrangement of computer fan array used to create the enhanced airflow (airflow+) treatment in lettuce.

Fig. S2. Spatial distribution of horizontal airflow velocity at 10 cm height in lettuce compartments under airflow– and airflow+ conditions.

Fig. S3. Temporal stability of baseline chamber airflow measured every 5 min over 14 d using a 2D sonic anemometer.

Fig. S4. Spatial distribution of PPFD within lettuce growth compartments.

Fig. S5. Spectral distribution of PPFD emitted by the Philips GreenPower LED modules used in the experiment.

Fig. S6. Air temperature, humidity, and CO_2_ conditions inside the gas exchange cuvette during VPD step changes for abaxial and adaxial measurements.

Fig. S7. Growth dynamics of the third leaf in dwarf tomato under airflow– and airflow+, shown as projected leaf area over time.

Fig. S8. Whole-plant water loss dynamics of lettuce under airflow– and airflow+, measured gravimetrically during day and night.

Fig. S9. Leaf-level gas exchange snapshots (*A*, *g*_sw_, iWUE) in dwarf tomato under airflow– and airflow+.

Fig. S10. Modelled stomatal conductance responses of adaxial and abaxial leaf sides to a step increase in VPD in lettuce grown under airflow– and airflow+.

Fig. S11. Representative transverse lettuce leaf cross-section showing raw micrograph and segmented adaxial/abaxial regions.

Table S1. Daytime and night-time environmental conditions (temperature, vapour pressure, VPD, and thermal time) in lettuce under airflow– and airflow+.

Table S2. Above-canopy microclimate and leaf temperature of dwarf tomato under airflow– and airflow+.

Table S3. Accumulated thermal time (degree-days) in lettuce calculated using heart temperature during the photoperiod.

Table S4. Growth and fruit traits of dwarf tomato under airflow– and airflow+, including leaf number, plant height, and fruit characteristics.

Protocol S1. Image processing and growth curve fitting protocol used to quantify lettuce canopy ground coverage from top-view images.

Protocol S2. Image processing protocol for segmenting lettuce leaf cross-sections and quantifying airspace.
