# Supplementary material for: Low wind, high impact: sustained low airflow alters water use efficiency and shapes plant acclimation
Source: J Exp Bot. 2026 Apr 17;77(16):5225–43. doi: 10.1093/jxb/erag185 (PMC13529328; doi:10.1093/jxb/erag185)
Supplement: Supplementary file 1 [file erag185_supplementary_data.pdf]

Supplementary Fig. S1. Fan array used to generate airflow+ in lettuce

Arrangement of the computer fan array used to create the enhanced-airflow (airflow+) treatment in lettuce.

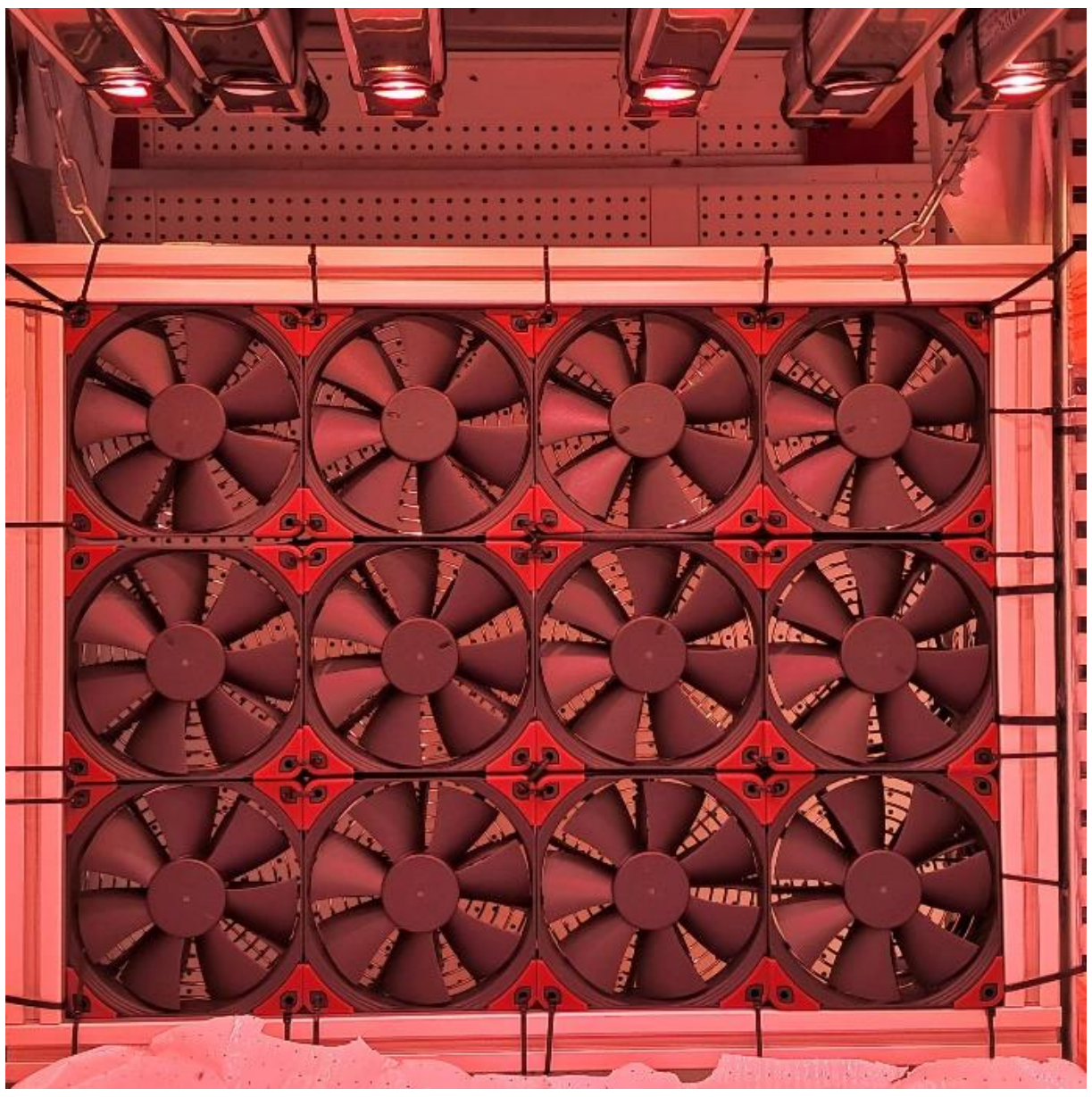

Supplementary Fig. S2. Spatial airflow distribution in lettuce growth compartments

Top-view maps of horizontal airflow velocity (m s^{-1}) measured at 10 cm height in low-airflow (airflow-) and enhanced-airflow (airflow+) compartments, showing spatial uniformity across plant positions.

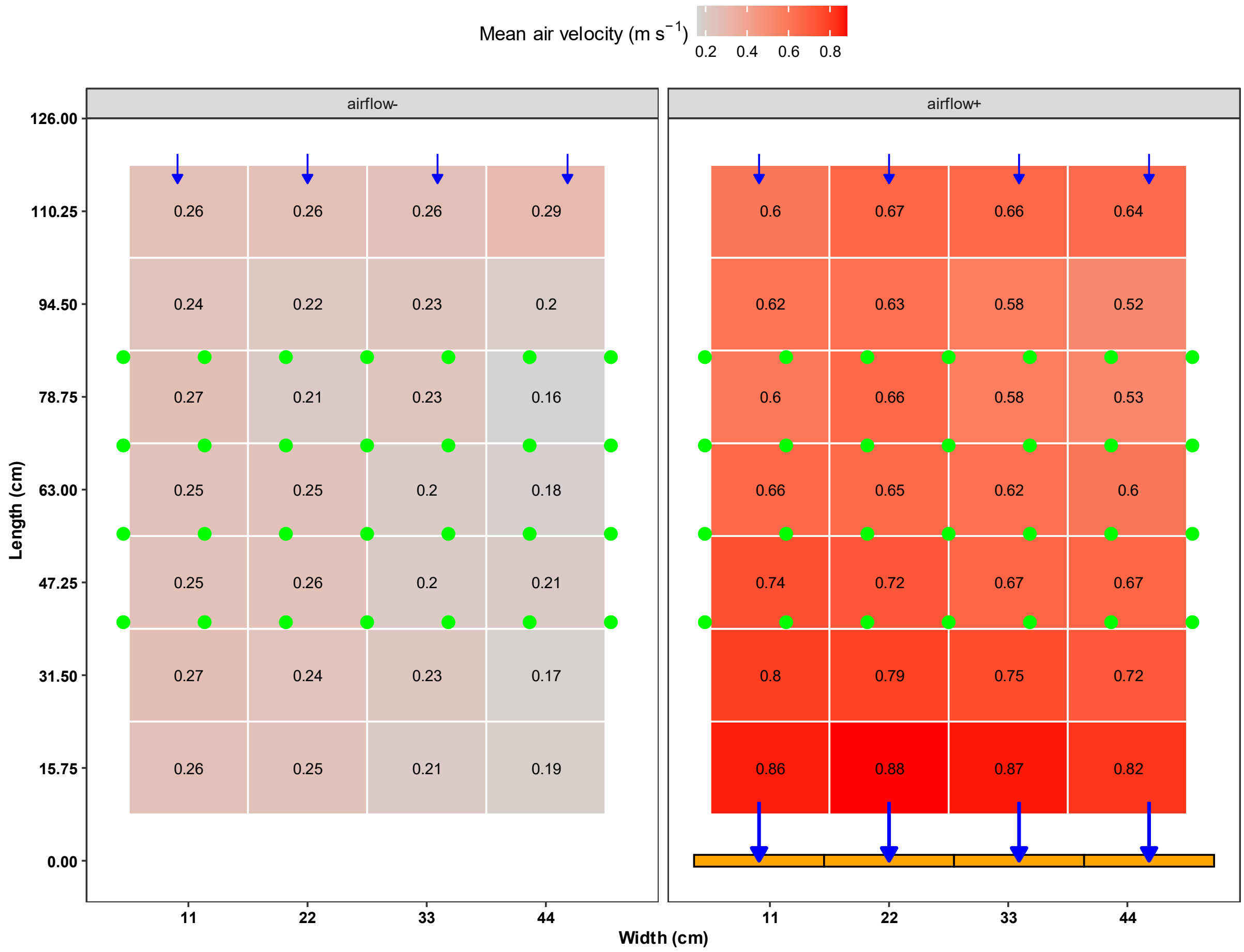

Supplementary Fig. S3. Temporal stability of chamber airflow

Horizontal airflow velocity measured every 5 min over 14 days using a 2D sonic anemometer, showing stable baseline airflow in the climate chamber; the LOESS-smoothed trend is shown in blue.

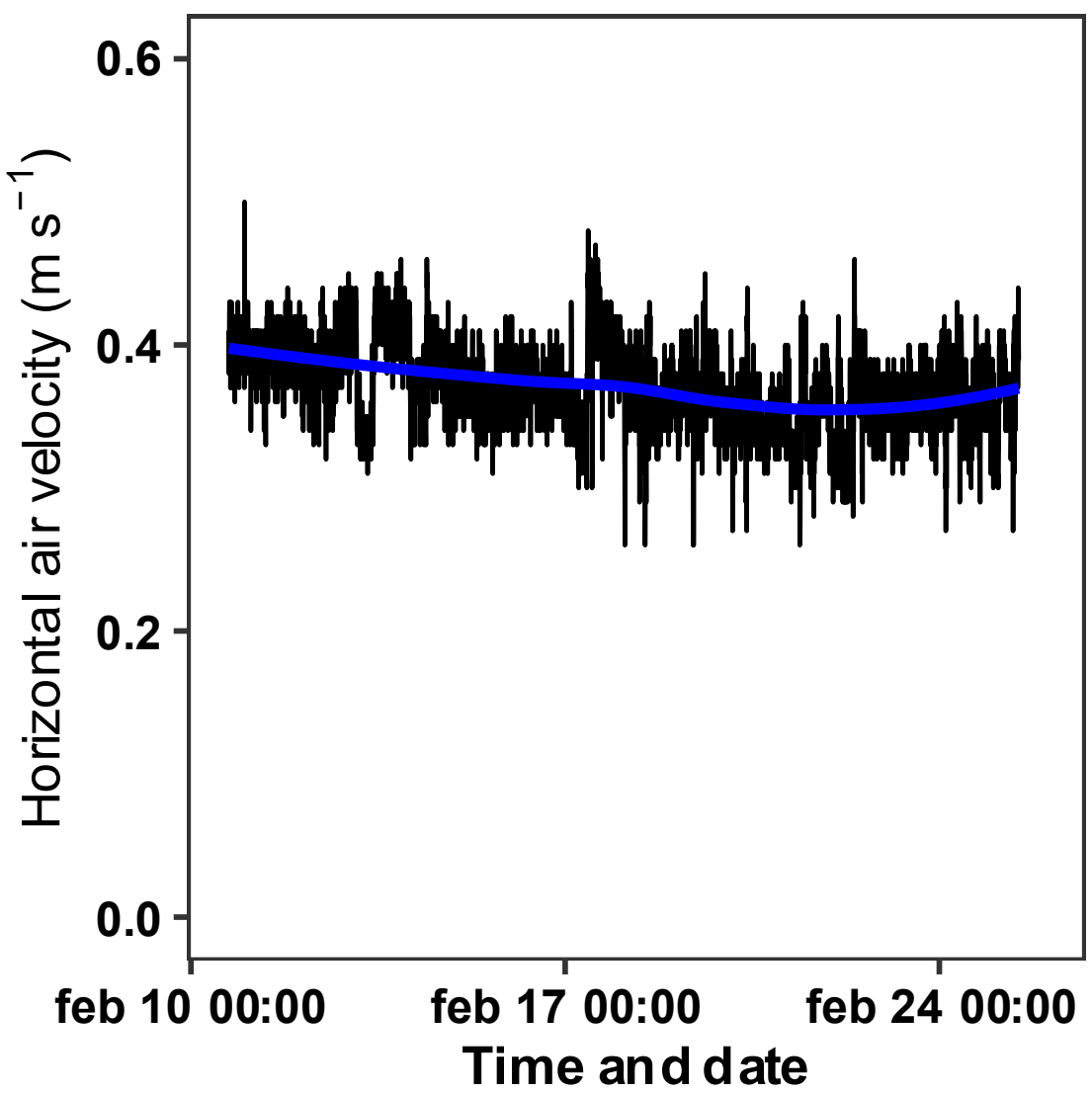

Supplementary Fig. S4. Spatial distribution of PPFD in lettuce compartments

Spatial distribution of photosynthetic photon flux density (PPFD) measured at 6.5 cm height across the 125 x 55 cm growth compartments, showing uniform light distribution (mean 178 μmol m^{-2} s^{-1}; variation between repetitions ~2.5%).

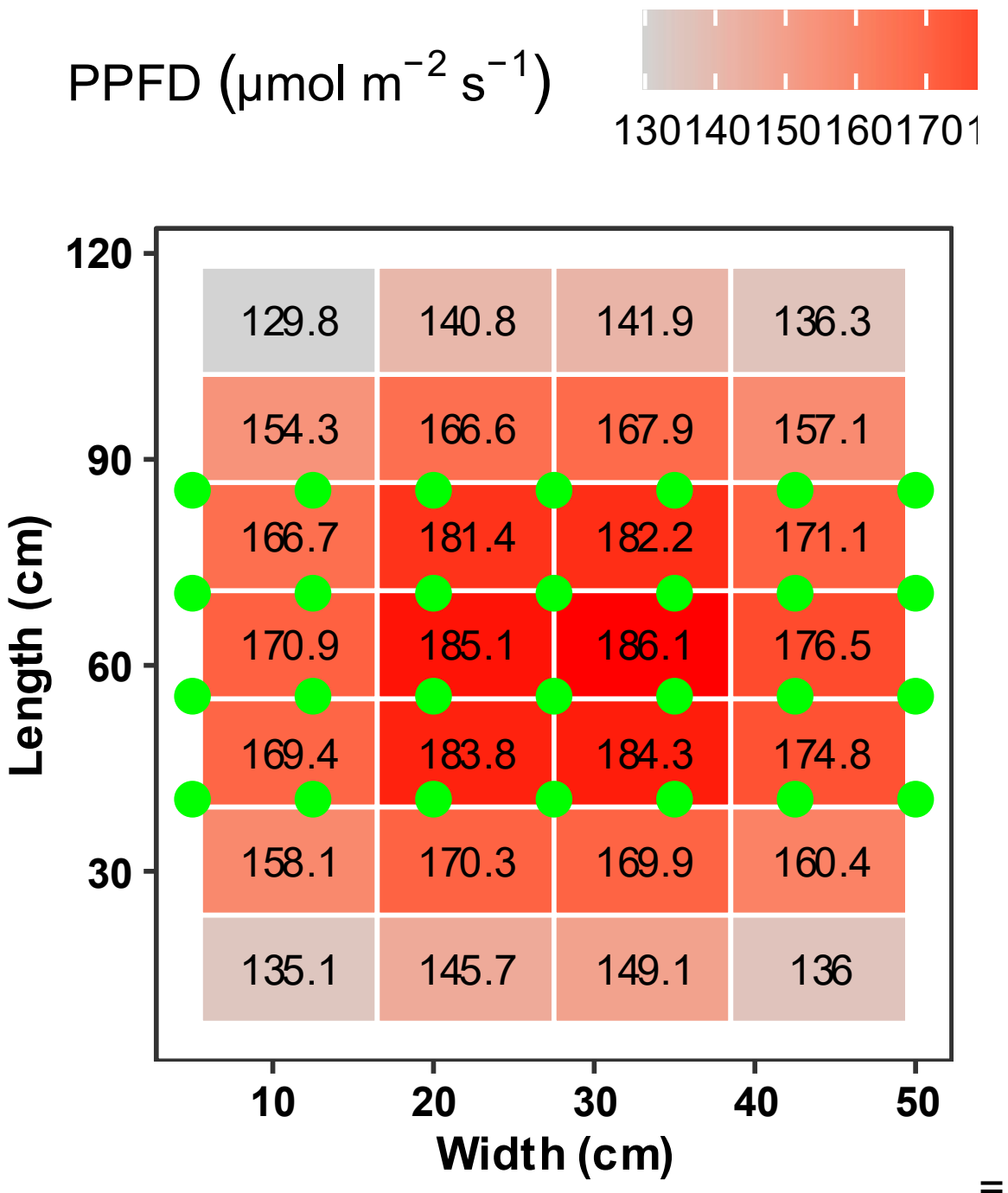

=

Supplementary Fig. S5. Spectral distribution of LED lighting

Spectral distribution of PPFD (per nm) emitted by the Philips GreenPower LED DR/W 120 LB modules used in the experiment.

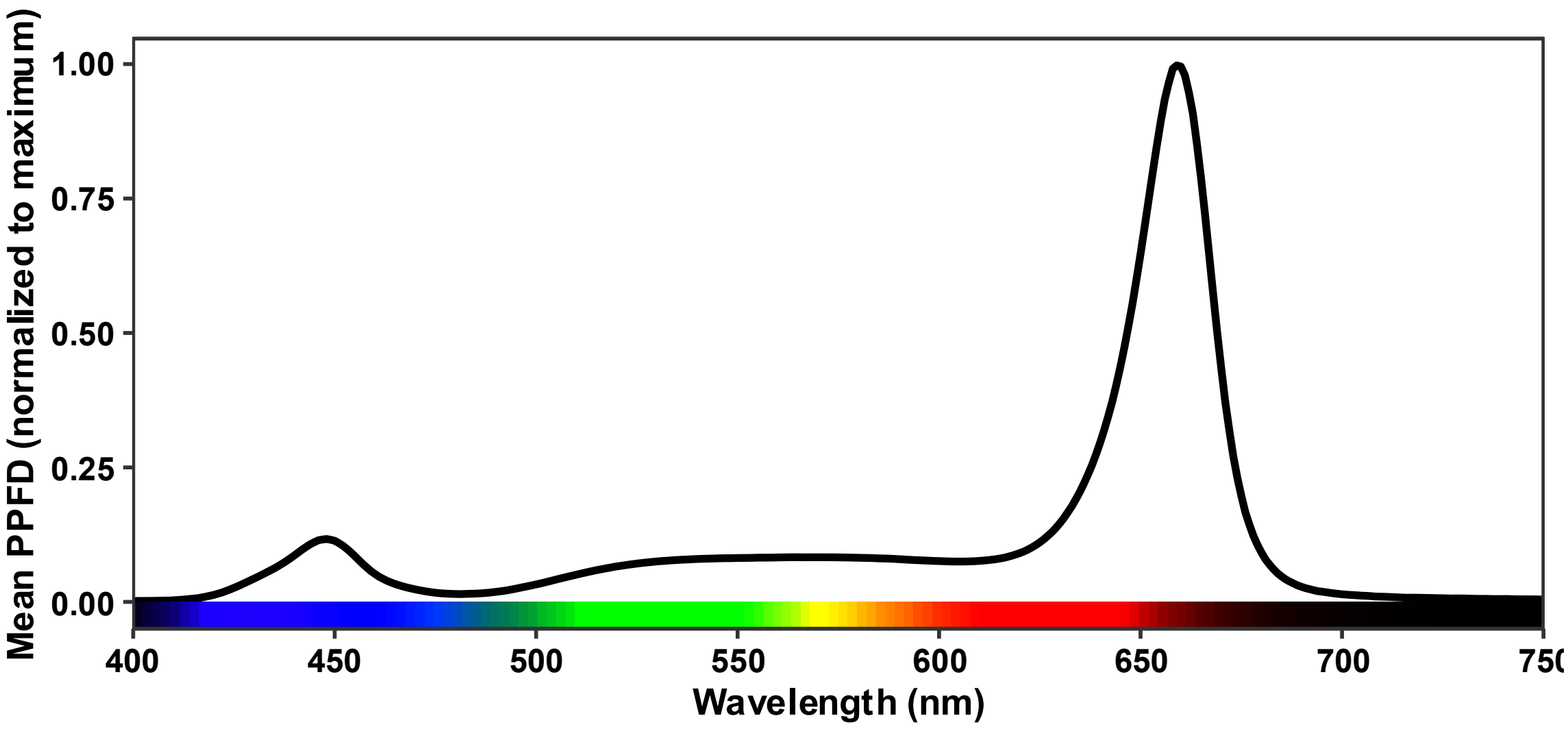

Supplementary Fig. S6. Air conditions during VPD step changes in the gas-exchange cuvette

Air temperature, relative humidity, and CO_2 concentration inside the portable gas-exchange cuvette during step changes in VPD for abaxial and adaxial leaf measurements of plants grown under low (airflow-) and enhanced (airflow+) airflow.

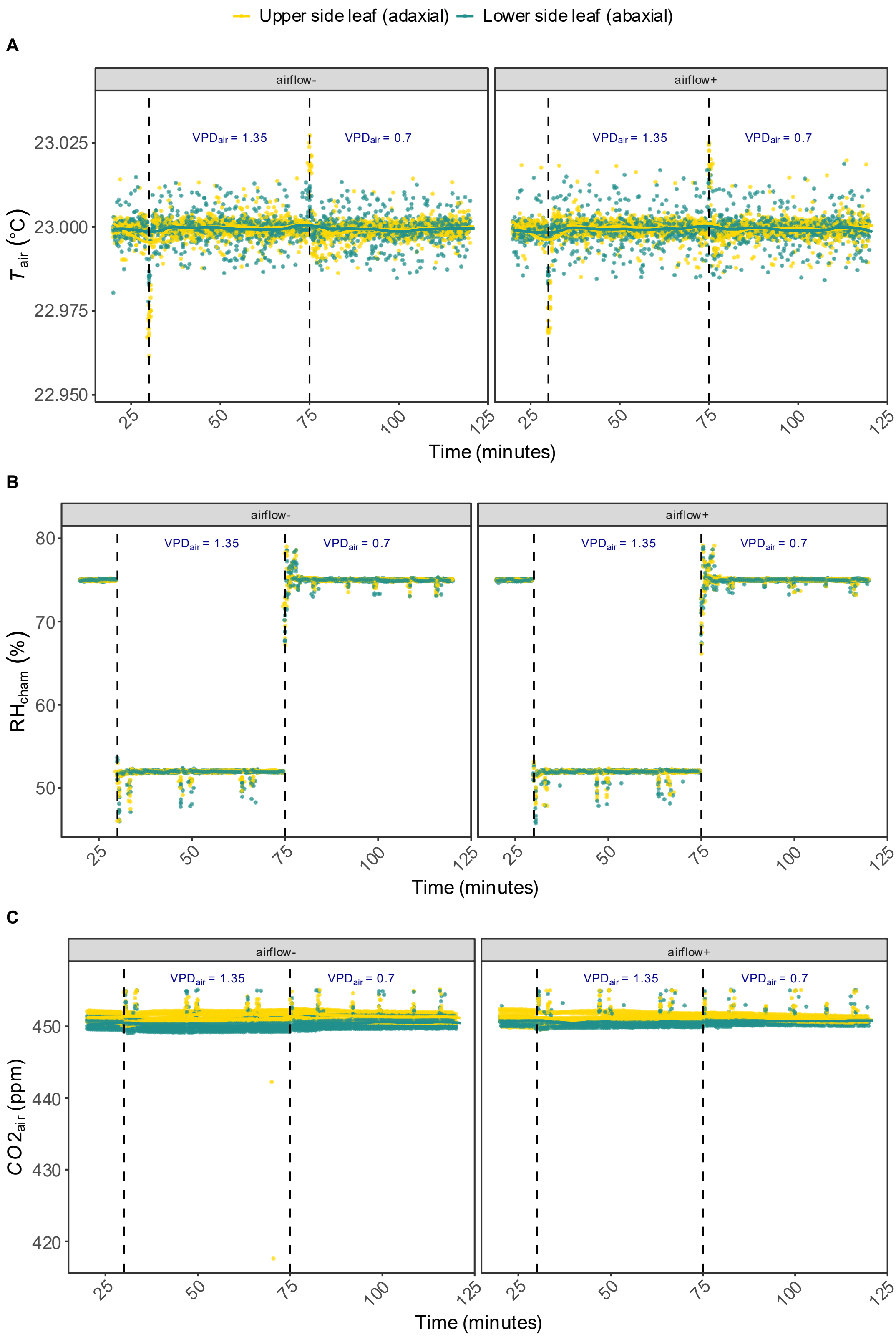

Supplementary Fig. S7. Growth dynamics of the third dwarf tomato leaf under airflow- and airflow+

Projected area of the third leaf over time for plants grown under low (airflow-) and enhanced (airflow+) airflow, shown as treatment means ± SE (n=6) with LOESS smoothed curves to illustrate temporal growth effects.

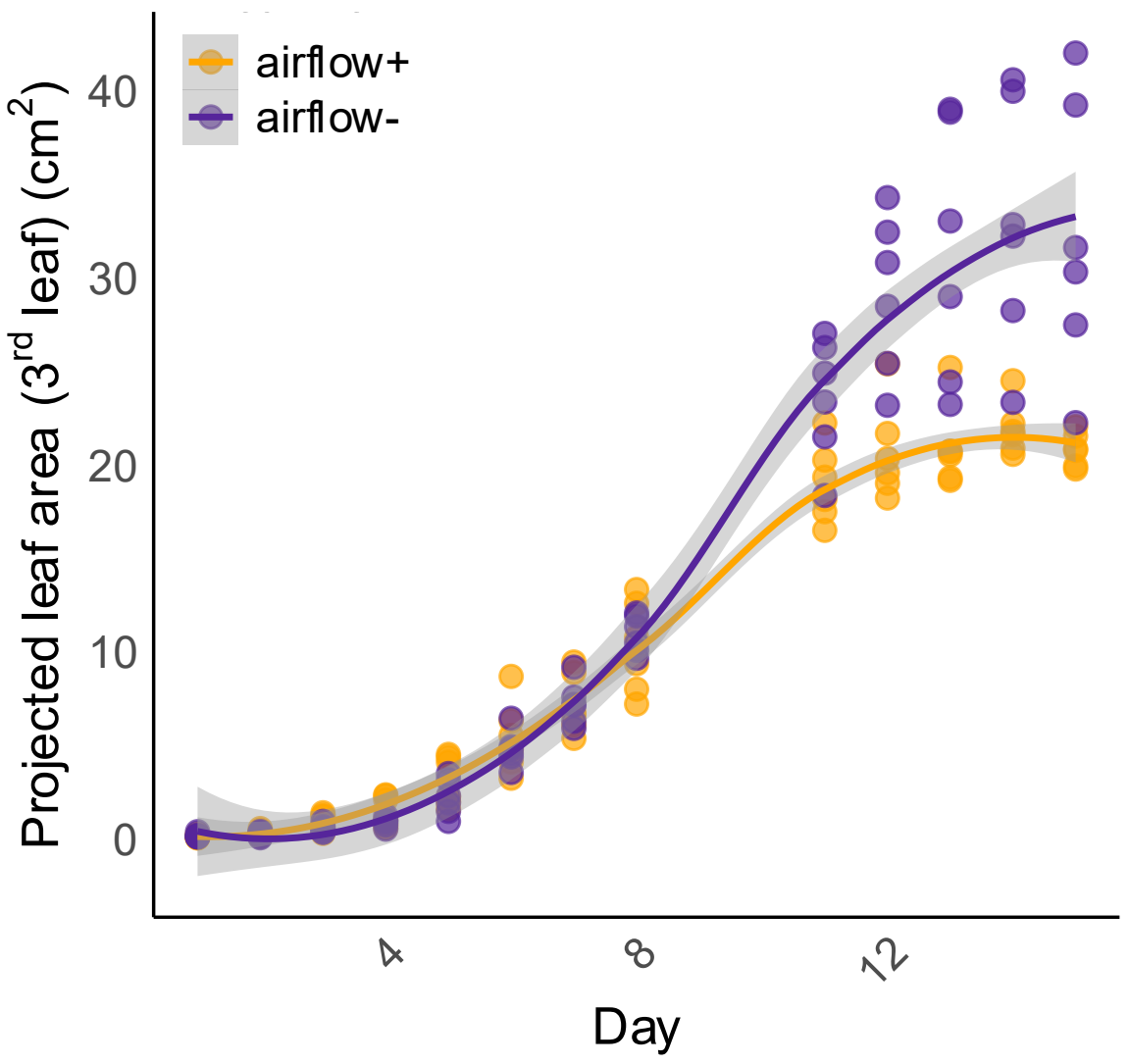

Supplementary Fig. S8. Whole-plant lettuce water-loss dynamics under airflow treatments

Whole-plant water-loss dynamics measured gravimetrically during daytime and nighttime in plants grown under low (airflow-: 0.22 m s^{-1}) and enhanced (airflow+: 0.65 m s^{-1}) airflow conditions; each line represents an individual plant's cumulative weight-loss trajectory across the measurement period.

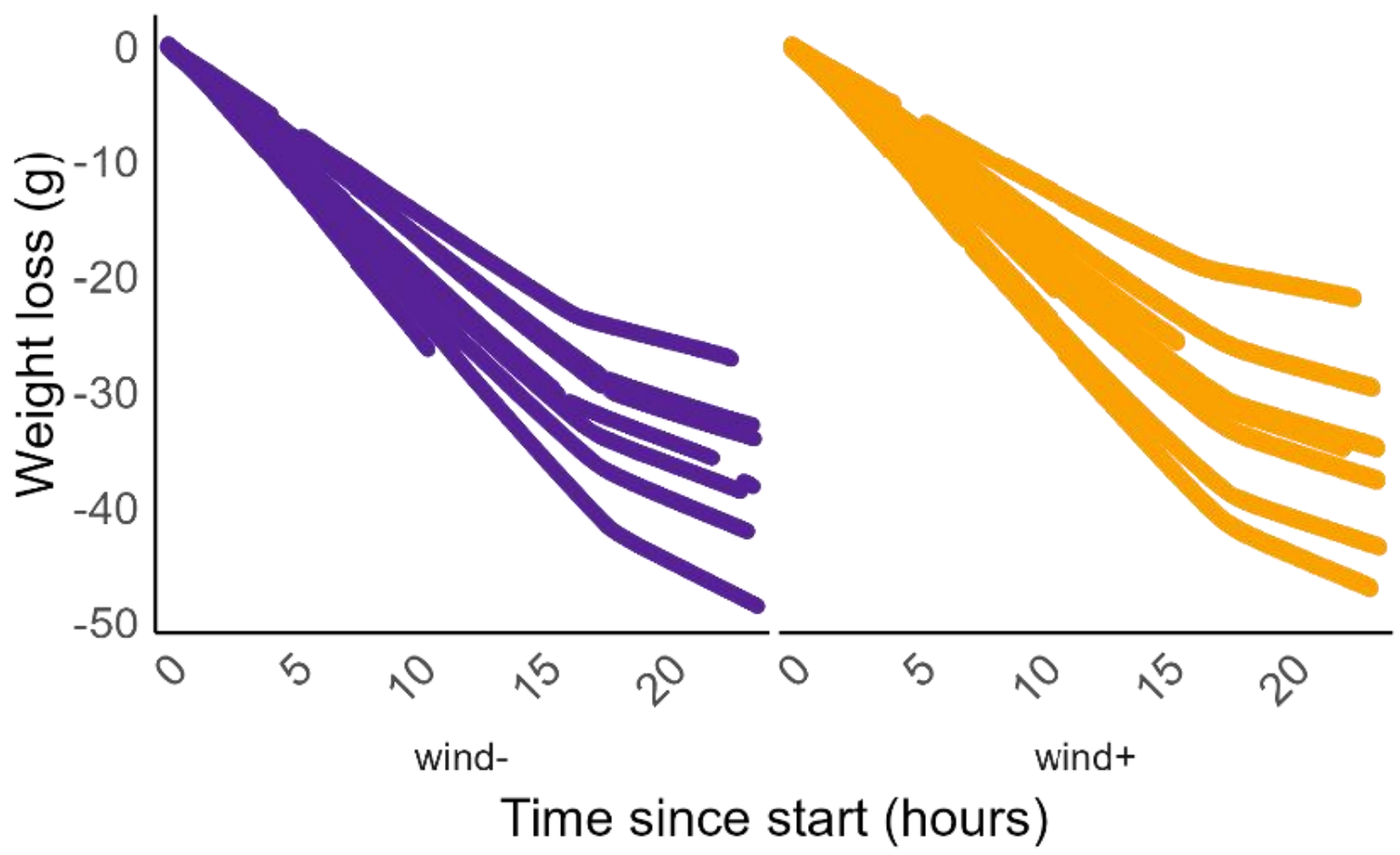

Supplementary Fig. S9. Leaf-level gas exchange under airflow- and airflow+ in dwarf tomato

Daytime net photosynthesis (*A*), stomatal conductance (g_{sw}), and intrinsic water-use efficiency (*iWUE*) snapshots measured 35 DAT under low (airflow-) and enhanced (airflow+) airflow, shown

as treatment means ± SE (n = 6); statistical differences were assessed using linear models with treatment and room side as fixed effects (type II ANOVA).

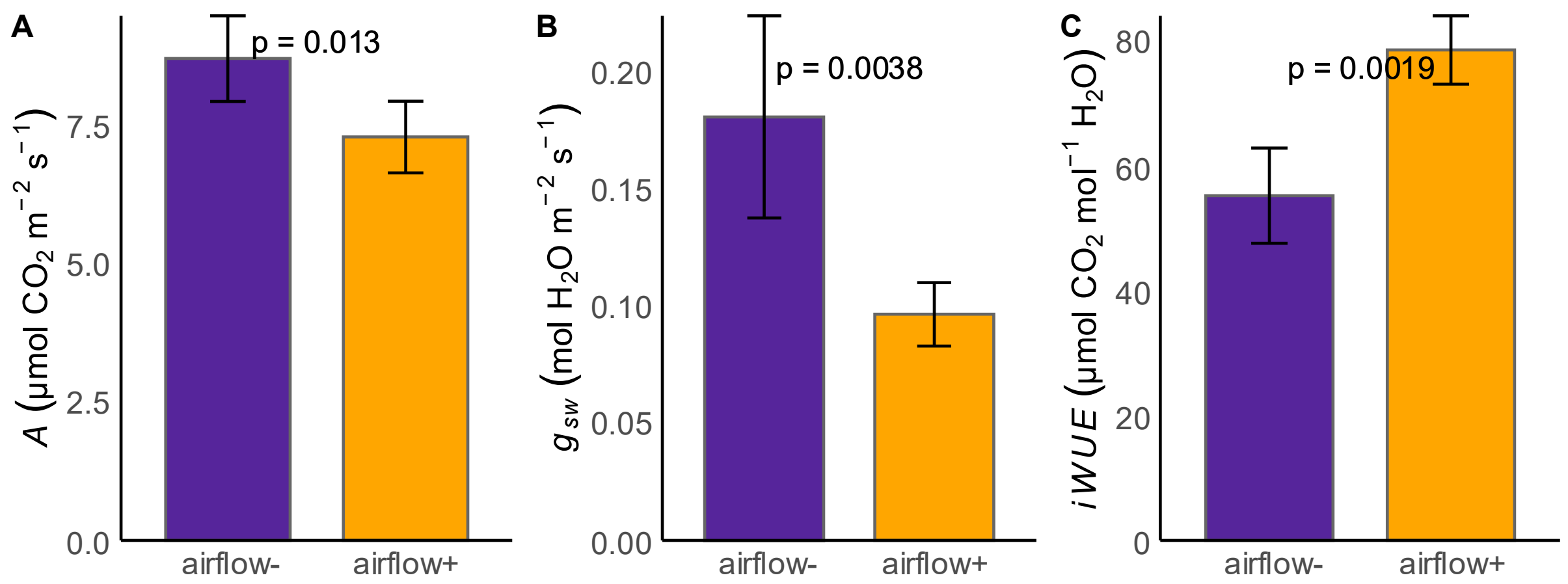

Supplementary Fig. S10. Stomatal conductance response to a step change in VPD

Modelled tomatal conductance (g_{sw}) of adaxial and abaxial leaf sides in lettuce plants grown under low (airflow-: 0.22 m s^{-1}) and enhanced (airflow+: 0.65 m s^{-1}) airflow following a step increase in VPDair from 0.70 to 1.35 kPa.

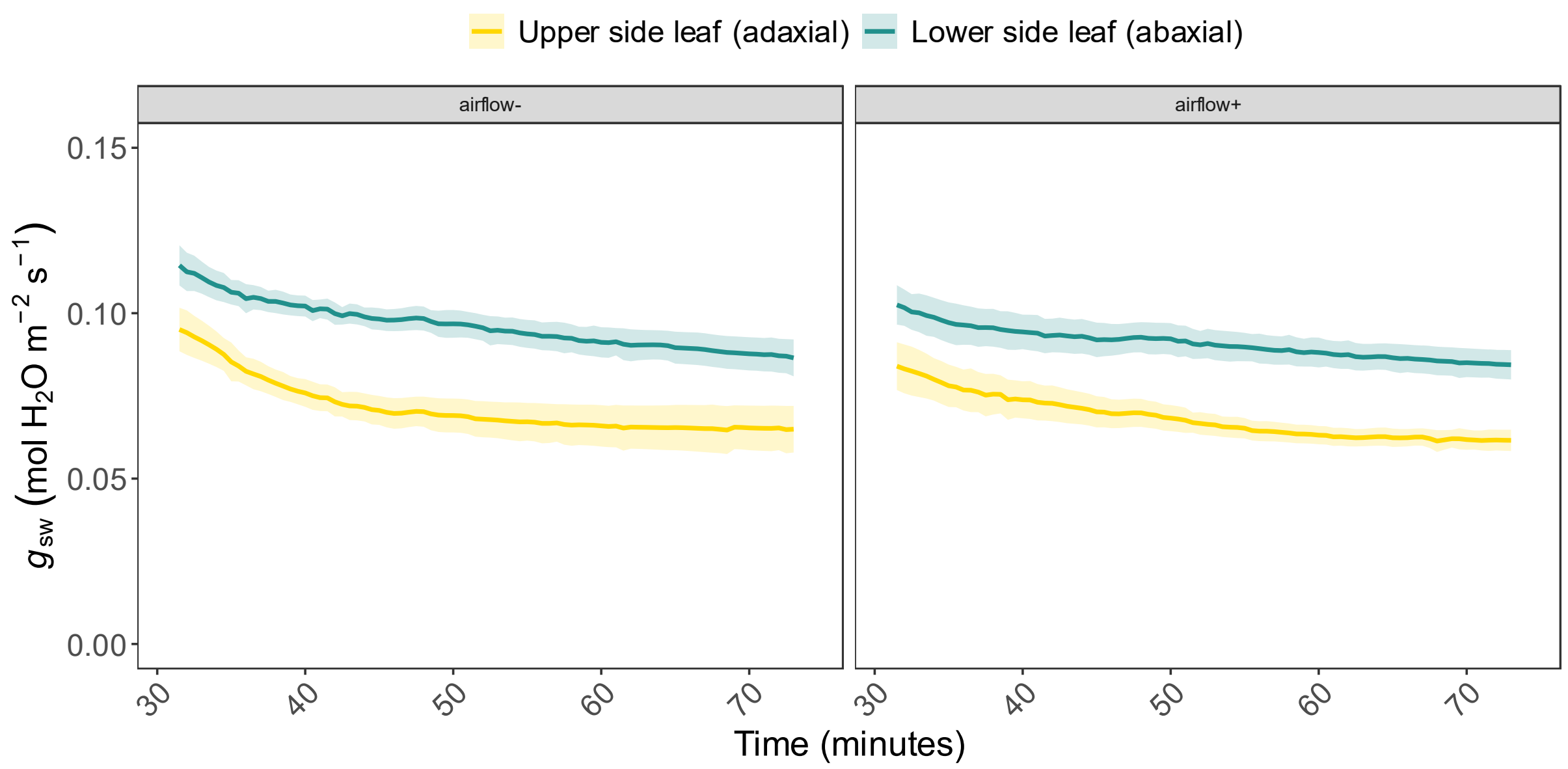

Supplementary Fig. S11. Transverse lettuce leaf cross-section

Transverse cross-section (10 μm) of a representative lettuce leaf at 25x magnification showing the white-balanced raw micrograph (A) and the segmented tissue region illustrating the automatically identified adaxial (orange) and abaxial (green) sides, with the midline (blue) separating both regions (B) with scale bar = 100 μm.

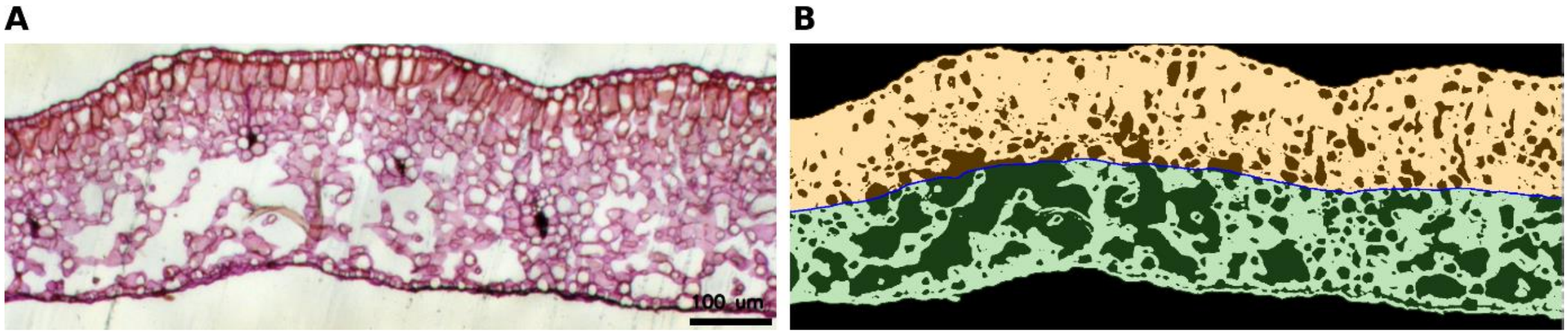

Supplementary Table S1. Environmental conditions in lettuce under airflow- and airflow+

Daytime and nighttime air temperature, vapour pressure deficit (VPD) vapour pressure (e_a), and thermal time (HU) under airflow- and airflow+ treatments; values are means ± SE (n = 6), with differences tested using paired T-tests.

Variable	Period	Airflow- mean	Airflow- SE	Airflow+ mean	Airflow+ SE	Difference	t	df	p
**e_a (Pa)**	Day	2107	3.930	2087	7.2	20.5	3.170	5	0.025
**e_a (Pa)**	Night	1806	5.260	1805	6.13	1.11	0.683	5	0.525
**Temp (°C)**	Day	23.1	0.185	22.7	0.149	0.439	3.330	5	0.021
**Temp (°C)**	Night	20	0.056	19.9	0.037	0.028	1.150	5	0.303
**VPD (kPa)**	Day	0.737	0.030	0.683	0.024	0.054	3.210	5	0.024
**VPD (kPa)**	Night	0.536	0.013	0.533	0.011	0.003	1.170	5	0.294
**HU d^{-1}**	n.a.	18.63	0.16	18.25	0.13	0.37	3.278	5	0.022
**HU (total)**	n.a.	493.6	4.34	483.8	3.49	9.85	3.278	5	0.022

Supplementary Table S2. Environmental conditions in dwarf tomato under airflow- and airflow+

Daytime above-canopy microclimate (5 cm above the meristem) and leaf-surface temperature for plants grown under low (airflow-) and enhanced (airflow+) airflow conditions; values represent means ± SE (n=6), with treatment and block effects tested by ANOVA.

Variable	Airflow - mean	Airflow se	Airflow+ mean	Airflow+ se	Difference	P(F) Treatment	P(F) block
Above-canopy microclimate							
**Airflow velocity (m s^{-1})**	0.210	0.049	1.03	0.016	-0.82	n.a.	n.a.
**ea (Pa)**	1994	16.7	1968	5.04	26.32	0.043	0.003
**Temp (°C)**	23.4	0.184	23.6	0.053	-0.225	0.152	0.016
**VPD (kPa)**	0.887	0.023	0.952	0.007	-0.065	0.02	0.23

Leaf microclimate							
**Leaf temperature**	22.25	0.37	23.09	0.27	-0.84	0.040	0.018

Supplementary Table. S3. Thermal time (degree days) calculated with lettuce heart temperature

Accumulated thermal time (HU) over 26.5 days for airflow- and airflow+ treatments, using lettuce heart temperature during the photoperiod with night air temperature.

T_base = 4 °C DAT = 26.5 days
With heart temperature during day, air temperature at night

**Treatment**	**Day temp (°C)**	**Night temp (°C)**	**Daylength (h)**	**Nightlength (h)**	**Avg. daily temp (°C)**	**HU (total)**	**HU d^{-1}**	**% diff**
Airflow-	22.55	20.00	18	6	21.91	474.68	17.91	
Airflow+	21.34	19.00	18	6	20.76	444.01	16.76	-6.46

Supplementary Table S4. Growth and fruit traits of dwarf tomato under airflow- and airflow+

Leaf number (20 DAT), plant height (44 DAT), and fruit traits (71 DAT) of dwarf tomato grown under low (airflow-) and enhanced (airflow+) airflow; values are means ± SE (n=6), with treatment and block effects tested using type II ANOVA.

	**Leaf number (# plant^{-1})**	**Plant height (cm)**	**Fruit FW (g)**	**Fruit length (cm)**	**Fruit width (cm)**	**Fruit DW (g)**
Airflow-	5.71 ± 0.12	19.12 ± 0.70	6.89 ± 0.17	24.86 ± 0.37	21.39 ± 0.30	523.84 ± 27.54
Airflow+	5.60 ± 0.08	17.94 ± 0.75	6.35 ± 0.17	24.52 ± 0.34	20.62 ± 0.29	495.75 ± 20.87
**Diff. (%)**	-1.93%	-6.15%	-7.73%	-1.37%	-3.56%	-5.36%
**Diff.**	0.11	-1.175	-0.53	-0.34	-0.76	-28.09
Diff 95 % CI	-0.453, 0.233	-1.12, 3.47	-0.006, 1.07`	-0.791, 1.48	0.208, 1.52	-51, 107
P-value treatment	0.49	0.28	0.05	0.51	0.02	0.44
P-value side room	0.61	0.32	0.30	0.32	0.01	0.42

Supplementary Protocol. S1. Image processing and growth-curve fitting for lettuce ground coverage

Overview: This protocol describes the step-wise image-processing workflow and growth-curve fitting used to quantify lettuce canopy ground coverage from top-view images

Image processing and data cleaning: Top-view images of the lettuce canopy were analysed using a custom Python script (Python 3.10.9; OpenCV, NumPy, pandas).

1) Images were imported and rectangular regions of interest (ROIs) were defined by manually selecting plant centres.
2) ROI boundaries were then generated automatically based on plant spacing, extending 0.5× the average horizontal spacing to each side and 0.5× the vertical spacing above and below the outer rows to ensure full coverage of each plant and its associated ground area.
3) Weekly manual cutouts were drawn on the final image of each week and applied to all images from that week to exclude areas where plants never grew. These cutouts removed artefacts such as algae and served as a reference to verify segmentation accuracy.
4) Leaf area was segmented using HSV colour thresholds to identify green tissue, with additional saturation filtering.
5) Segmented masks were refined using the ROI, exclusion zones, and weekly cutouts.
6) Age-dependent morphological operations (e.g. erosion, dilation, minimum contour area filtering) were applied to remove early-stage noise and non-leaf artefacts.
7) For each image, the number of pixels classified as lettuce within the ROI was counted, and ground coverage was expressed as the percentage of the ROI occupied by leaf tissue.

Growth-curve fitting:

8) Average measurements from multiple cameras within each replicate to obtain a single value per treatment x replicate x timepoint.
9) Fit ground-coverage time series using the Richards functions, with the parameter Q fixed at 2.5 to improve fit stability and reduce collinearity:

$$Ground\ coverage\ (t) = \frac{K}{(1 + e^{-C(t-t_i)})^{1/Q}}$$

10) Interpret parameters with K the asymptote and thus maximal ground coverage, C the growth rate, and t_i the inflection point

Supplementary Protocol. S2. Image processing of lettuce leaf cross-sections

Overview: This protocol describes the image-processing workflow used to segment leaf lamina cross-sections, assign adaxial/abaxial orientation, and quantify airspace and thickness from microscope images.

Image import and preprocessing: Leaf cross-section images (.czi, Zeiss format) were analysed using a custom Python script (Python 3.10.9; libraries: numpy, opencv-python, czifile, matplotlib, pandas, scikit-image, and SciPy).

1) Images were imported and converted from RGB to CIELAB colour space.

2) The A channel (red–green component) was extracted to increase tissue contrast and Gaussian-blurred (5x5 kernel) to reduce noise.
3) Leaf tissue was segmented using Otsu's thresholding, followed by morphological closing to obtain a continuous lamina mask.
4) The largest contour was identified as the leaf lamina, converted to a filled binary mask and rotated using the minimum-area bounding rectangle so that the lamina's long axis was horizontal.
5) A fixed proportion of the distal ends of the lamina was cropped from both sides to avoid edge artefacts caused by non-straight cuts.
6) For each column within the rotated lamina mask, the upper and lower part was detected to form a curved midline.
7) Within each half, the airspace was calculated as the proportion of non-tissue pixels (i.e. non coloured) relative to all pixels inside the leaf mask and expressed as percentage airspace. The abaxial/adaxial orientation was assigned automatically, presuming that the abaxial side has higher airspace percentage.
8) All assignments were visually verified by overlaying the lamina mask, midline, and labelled adaxial/abaxial regions.
9) Pixel distances were converted to micrometres using microscope-specific calibration factors (25× = 1.38 μm px^{-1}, 50× = 0.69 μm px^{-1}, 80× = 0.43 μm px^{-1}).

Each image was manually reviewed and accepted results were exported as a CSV summary file containing metadata and thickness.
